# Exploring the Dependence of Spectral Properties on Canopy Temperature with Ground-Based Sensors: Implications for Synergies Between Remote-Sensing VSWIR and TIR Data

**DOI:** 10.3390/s25030962

**Published:** 2025-02-05

**Authors:** Christos H. Halios, Stefan T. Smith, Brian J. Pickles, Li Shao, Hugh Mortimer

**Affiliations:** 1 School of Built Environment, University of Reading, Whiteknights, Reading RG6 6DF, UK; s.t.smith@reading.ac.uk (S.T.S.); l.shao@reading.ac.uk (L.S.); 2 School of Biological Sciences, University of Reading, Whiteknights, Reading RG6 6EX, UK; b.j.pickles@reading.ac.uk; 3 Rutherford Appleton Laboratory, Harwell Campus, Didcot OX11 0QX, UK; hugh.mortimer@stfc.ac.uk

**Keywords:** thermal measurements, spectral reflectance, synergy, vegetation canopy, absorption features, remote sensing, PROSAIL, carotenoids, *LAI*

## Abstract

Spaceborne instruments have an irreplaceable role in detecting fundamental vegetation features that link physical properties to ecological theory, but their success depends on our understanding of the complex dynamics that control plant spectral properties—a scale-dependent challenge. We explored differences between the warmer and cooler areas of tree canopies with a ground-based experimental layout consisting of a spectrometer and a thermal camera mounted on a portable crane that enabled synergies between thermal and spectral reflectance measurements at the fine scale. Thermal images were used to characterise the thermal status of different parts of a dense circular cluster of containerised trees, and their spectral reflectance was measured. The sensitivity of the method was found to be unaffected by complex interactions. A statistically significant difference in both reflectance in the visible (VIS), near-infrared (NIR), and shortwave infrared (SWIR) bands and absorption features related to the chlorophyll, carotenoid, and water absorption bands was found between the warmer and cooler parts of the canopy. These differences were reflected in the Photochemical Reflectance Index with values decreasing as surface temperature increases and were related to higher carotenoid content and lower Leaf Area Index (*LAI*) values of the warmer canopy areas. With the increasingly improving resolution of data from airborne and spaceborne visible, near-infrared, and shortwave infrared (VSWIR) imaging spectrometers and thermal infrared (TIR) instruments, the results of this study indicate the potential of synergies between thermal and spectral measurements for the purpose of more accurately assessing the complex biochemical and biophysical characteristics of vegetation canopies.

## 1. Introduction

Remotely sensed spectral measurements have proven to be valuable in vegetation-related research with applications in biodiversity conservation [1], agriculture [2], forestry [3], urban green infrastructures [4], and other related fields [5]. Spectral characteristics of vegetation have been long studied at both the leaf and canopy levels in forests, agricultural crops, and urban areas. At the canopy level, spectral measurements have been conducted with sensors (e.g., spectroradiometers) deployed in a variety of platforms involving both field spectroscopy with in situ measurements ([6,7] and references therein) or remote sensing platforms with the sensors carried either on airplanes [8,9], unmanned aerial vehicles [10], or on satellites [11,12,13].

Remotely sensed spectral measurements and, in particular, data from spaceborne instruments have an irreplaceable role in relating spectral and thermal observations of vegetation to landscapes and regions [14] and in detecting fundamental vegetation properties that link physical properties to ecological theory [15] in the field of terrestrial ecology. With 90 named instruments for scientific and/or environmental studies on 61 Earth Observation (EO) satellites in orbit as of the start of 2024 [13], large streams of data from land monitoring spaceborne instruments are becoming available, with increasingly improved spectral [16] and spatial resolution [17], offering synergies between spectral reflectance and thermal data [18]. However, the success of these approaches depends ultimately on our understanding of the complex dynamics that control plant spectral properties and our ability to accurately interpret reflectance data [14]. This effort faces challenges because of the spatial heterogeneity of remotely sensed fields at the fine scale, resulting in mixed pixels; this is particularly evident in complex environments such as, for example, urban areas where the spatial resolution of these sensors renders them suboptimal for urban land-cover classification or the study of green infrastructure [18]. To tackle this, ground-based surveying and sampling (providing points of reference) are required: even though this is a labor-intensive, time-consuming task only to be limited to small scales [19], it is nevertheless necessary for the validation of remotely sensed datasets acquired.

Another significant challenge results from the leaf- and canopy-level effects being complicated by complex inter-relationships among vegetation features and environmental parameters. The combined effect of this complexity affects the spectral reflectance measured at the top of the canopy. For example, the interdependence of canopy surface temperature and the spectral reflectance at leaf and canopy level has been studied little. The literature on the combined use of thermal cameras and spectrometers in the context of studying the following is extensive: plant water status [20] and evapotranspiration monitoring and modelling [21]; plant health [22,23]; early detection of heat stress [24,25]; plant environmental interactions, in terms of stomatal conductance to water vapour or transpiration, with various applications in agronomy, ecology, environmental sciences, and also in the agri-food industry [26]; or general vegetation assessment [27]. Some of these studies have used data from spaceborne instruments as early as the mid-eighties [27]. Recently, some studies have reported coupling of multi- or hyper-spectral measurements with thermal imagery from satellites for improving the spatial resolution of Land Surface Temperature (LST) maps [28]; compositional mapping by integrating data from the VNIR, SWIR, and LWIR spectral ranges [29]; or evapotranspiration estimation [30]. In most studies, thermal and optical data are used in a complementary way, with only a few studies attempting thermal–optical integration and then only at fine spatial scales (e.g., individual leaves) not captured by remote sensing instruments [31,32]. Thus, interactions between thermal and spectral reflectance properties and their dependence on the vegetation’s biophysical and biochemical characteristics remain largely unexplored.

The aim of this study was to present a ground-based experimental layout that enables synergistic thermal and spectral measurements to be collected for the purpose of assessing top-of-the-canopy reflectance at the fine scale. The data collected were used to explore differences between the warmer and cooler areas of the canopy. This study will provide a valuable resource and methodology to complement airborne/satellite sensor studies and/or for calibration and ground-based validation of other datasets—especially in cases where spatial heterogeneity imposes challenges in the interpretation of remotely sensed data.

## 2. Materials and Methods

### 2.1. Instrumentation

An SM2500 spectrometer (Spectral Evolution, Lawrence, MA, USA; nominal spectral resolution of 3.5 nm in the range 350–1000 nm and 22 nm at 1500–2400 nm, wavelength accuracy is 0.5 nm) was deployed coupled with a newly calibrated 10 m fibre optic cable (25° Field Of View–FOV, Spectral Evolution Lawrence, MA, USA) in order to measure crown level reflectance within the range of 350–2500 nm. Spectra were output for 768 wavelengths. The spatial resolution of the reflectance measurements used in this study ranged between 1.16 m/picture and 1.55 m/picture.

A PI 640 infrared camera (Optris, Berlin, Germany; spectral range: 7.5–13 μm, horizontal FOV: 33°, vertical FOV: 25°) was used, giving pictures with an analysis of 640 × 480 pixels. The camera was equipped with a 10 m common USB cable for connection with the laptop (Dell, Inspiron). The interthermal picture ground sampling distance for measurements used in this study ranged between 0.78 m/picture and 1.55 m/picture, and the thermal camera’s resolution ranged between 1.6 mm/pixel and 2.45 mm/pixel (corresponding to sensor heights 1.75 m and 2.65 m above the mean canopy height). Following [33], a constant emissivity was used, assuming that the relative effect of the emissivity correction is small and can be ignored, and the temperature calculated is called “surface temperature”.

**Figure 1:**
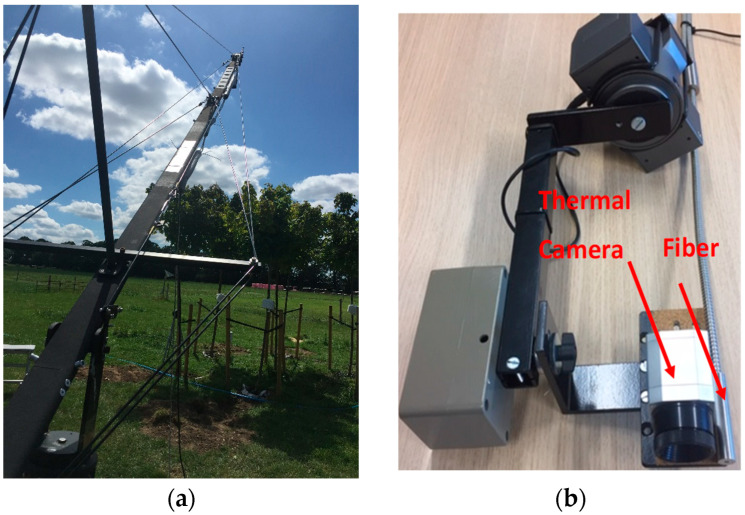
(**a**): The portable crane used for the top-of-the-tree canopy reflectance measurements. (**b**): Thermal camera and fibre optic attached on the powerhead.

The end point of the 10 m fibre and infrared camera was mounted on the plate of a movable Hague UPH Underslung 360° Pan & Tilt Camera Powerhead (Hague, Nottingham, UK); this was placed at the end of a Proaim Wave-5P 24ft Camera Jib Crane (Proaim, Zaventem, Belgium). The crane was standing on a tripod (W5-STD Stand) firmly placed on an aluminium trolley (D-37 Floor Dolly) with 360° rotating wheel bearing (Figure 1a). The entire mechanism (jib crane, tripod, and trolley) was movable. The height of the measurements was calculated based on measurements described in Appendix A.

The two sensors (camera and fibre optic) were mounted on the powerhead next to each other (Figure 1b), and the distance between the two instruments was ~2 cm. The FOVs of the spectrometer and the infrared camera were compared in the lab. The fibre optic of the spectrometer was connected to a light source and the resulting illuminating circle was measured to be the spectrometer’s FOV. The thermal camera’s FOV was calculated by marking the exact position of an approaching warm object. Ten measurements of the two FOVs were made with the instruments placed at a height of 55–58 cm above a level surface. After each measurement, the two instruments were dismantled and placed back anew on the powerhead before a new measurement was taken. Measurements showed that the common area captured by both instruments corresponded to 0.87 ± 0.07 (mean ± standard deviation) of the spectrometer’s FOV and to 0.76 ± 0.07 of the thermal camera’s FOV. A host of ancillary measurements were also collected and are described in Appendix B.

### 2.2. Experimental Site and Trees

During the period between 22 May and 25 September 2019, seven containerised maples (*Acer platanoides* ‘Globosum’, Globe Norway Maple) were placed within the 580-hectare Shinfield Farm of the University of Reading (51°24′45.4″ N 0°54′39.4″ W).

These trees were chosen because they naturally form a uniform, dense, and almost spherical crown, minimising gaps in the tree canopy when clustered in a circular arrangement. The heights of the trees were measured on 25 September to be 3.4 ± 0.1 m (mean ± standard deviation); the diameter of the canopies was 2.0 ± 0.3 m, and their vertical extent was 1.4 m ± 0.2 m.

Each tree was placed in a cylindrical container (40 cm diameter × 40 cm depth—provided by Barcham Trees PLC) with the tree trunk strapped on two wooden posts affixed to the ground for this specific purpose. For extra stability, the containers were fitted in rectangular holes opened in the ground using shovels and then back-filled with soil. Irrigation was applied with the use of an automated system, typically programmed to operate three times a day (5:00, 13:00, and 21:00) for half an hour of irrigation. Irrigation duration and frequency varied through the entire experimental period depending on the general meteorological conditions. The water flow rate used for the irrigation was tested on 7 October, and the average was found to be 27.6 L h^−1^ per tree (*IQR* 8 L h^−1^); therefore, the average volume of water supplied to the trees in an irrigation period corresponds to ~14 L per tree.

**Figure 2:**
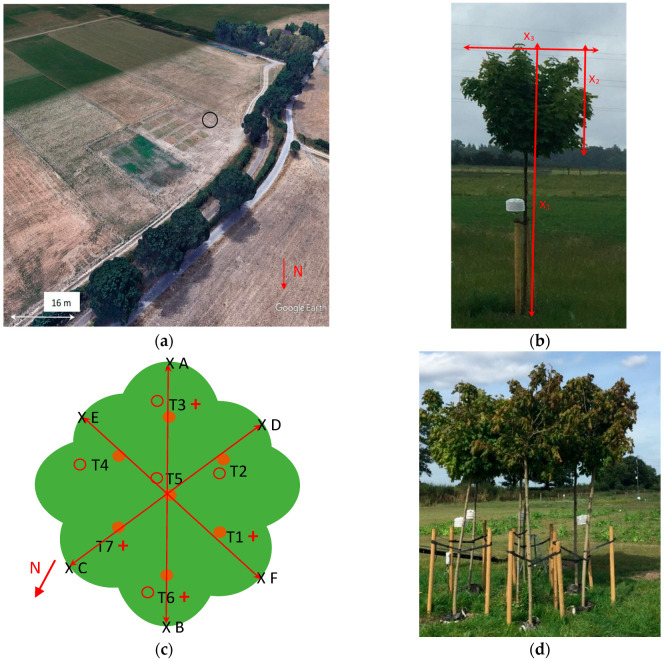
The location of the experimental site (Shinfield Farm of the University of Reading 51°24′45.4″ N 0°54′39.4″ W). The site is depicted with the circle (**a**). Tree T7 and the main dimensions discussed in text: height of the tree (x_1_), vertical extent (x_2_), and diameter of the canopy (x_3_) (**b**). Schematic representation of the tree cluster. Filled circles and *Ti* for *i* = 1:7 denote trees, and the names of the trees respectively; ‘x’ symbols show the location of the points A, B, C, D, E, and F. Distances AB, CD, and EF are 3 m, 3.10 m, and 2.60 m, respectively. Dimensions shown are not to scale. Open circles and crosses denote soil moisture and air temperature sensors, respectively (**c**). The tree cluster, as seen from northwest ($\sim 320^{{}^{\circ}})$ (**d**).

Measurements took place in a controlled environment consisting mainly of a grassy field (Figure 2); this ensured that the measurements were not influenced by reflections or thermal interactions from/with surrounding buildings or objects which are often found in other (e.g., urban) environments. Mature trees were situated in the WSW direction from the test site, and the closest one was at a distance of ~20 m from the experimental site. The grass around and close to the trees was mowed on a regular basis.

### 2.3. Experimental Protocol and Periods

Sets of canopy reflectance measurements and thermal images were obtained by first taking one reference reflectance reading at the reference plate, followed by multiple reflectance readings and thermal images for different samples of the tree cluster canopy. An 8 cm × 8 cm square Spectralon reflectance panel (provided by Field Spectroscopy Facility), horizontally placed on a tripod ~65 cm a.g.l., several meters away from the cluster of trees was used for reference reflectance readings; integration time of reflectance measurements was 10 ms for 10 scans per sample. Every reflectance reading was accompanied by a thermal image so the “source area” of every individual spectrum could be evaluated and compared (see Section 2.4.1). All measurements were conducted with the instruments mounted on the crane pointing downwards to the tree canopy, 2.5 h from solar noon, to control variations of the solar elevation and the solar azimuth. All measurements were conducted when direct solar radiation was not obstructed by clouds.

Within each set of spectrometric measurements, a range of (3–11) reflectance readings and thermal images at different samples of the tree cluster were taken. The reflectance readings and thermal images at different samples of the tree cluster canopy were obtained by changing the sensors’ azimuth by a few degrees (ranging between 3° and 10° within each set of measurements) so that samples from the entire canopy were obtained in one set. The sensors’ azimuth change (and, therefore, the number of readings taken within each set) depended mainly on the height of the sensors, i.e., when sensors were placed higher above the canopy, a wider range was captured within their FOVs, and therefore, fewer readings (i.e., fewer samples) would be required to cover the entire canopy. The time needed for the completion of one set of measurements depended on the number of spectrometric measurements obtained (i.e., the number of samples measured) and varied between 40 s and 160 s with corresponding range of measurement set (3–11). The number of measurements taken for every set had an average value of 5.4 across all sets and corresponded to an average value of 85 s (for 5 and 6 measurements per set).

During the period between 6 August and 25 September 2019, the trees were clustered in a near-circular arrangement. In this arrangement, trees’ canopies were in very close proximity, and minimum gaps were allowed between the individual foliage. The base of the crane was positioned ~3.70 m away from the tree placed at the SW edge of the canopy. Seen from this distance, the tree cluster canopy at ground level extended within an arc of ~30° (between 120° and 150°).

After the completion of one set of measurements, at least two more sets were conducted at the same height, completing one cycle of measurements for that height. Thus, each cycle of measurements consisted of at least nine reflectance readings and nine thermal images. More cycles of measurements were repeated, if deemed necessary, based on the specific conditions of the set(s), i.e., mainly the in situ visual inspection of the quality of the spectra (depending on the stability of light during the set).

The sensitivity of the method to changing the sensor’s height was tested by taking measurements on a cluster of trees: (i) first at multiple heights and then (ii) at two heights. The measurements from (i) were used to obtain a quick overview of the effect of changing the sensor’s height and to explore the parameters controlling spectral reflectance; measurements from two heights (ii) were used to test the spectral differences in detail. Applying a linear regression technique, spectral measurements taken at two different heights above the canopy were compared while the controlling parameters (accounting for meteorology, illumination conditions, and biophysical attributes) were kept unchanged. It was found that the effect of changing the height of sensors on reflectance measurements was negligible (overall spectral separability is <1%). Details are described in Appendix C.

Reflectance measurements of the surface background (grass and soil) were also taken on 29 July following the same methodology; 11 grass and 8 soil reflectance spectra were taken and were further processed and used during the modelling stage of the analysis (Section 3.3).

For the reflectance measurements at the leaf level, an attached leaf clip accessory (part of the SM 2500 spectrometer), equipped with an integrated Spectralon standard (Spectralon Labsphere, Inc., North Sutton, NH 03260, USA) for white referencing, was used before reflectance readings were taken (integration time of 10 ms for 10 scans per sample). Leaf samples were illuminated by an external light source (Spectral evolution ILM 105—Spectral Evolution Lawrence, MA, USA), and three spectral reflectance readings were taken on the adaxial leaf surface of each sample. Samples (leaves) were taken from the south-facing lower part of each tree. Reflectance measurements at leaf level were conducted on 18 July 2019 and 26 September 2019. In this paper, measurements taken on 18 July 2019 are discussed.

### 2.4. Post Processing

#### 2.4.1. Thermal Imaging Analysis

To account for the structural features of the canopies and illumination conditions that influence the measured reflectance spectra, the proportion of the cooler areas of the canopy to the warmer areas of the canopy (hereafter, AcAw) obtained from thermal imaging analysis were used. This parameter is an indicator of the shadowed and sunlit areas and is, therefore, related to both the biophysical attributes of the part of the canopy captured and illumination conditions in every thermal image. This will be further explored in Section 3.1.

The procedure by which this parameter has been calculated is described as follows:

From the original thermal image files, four variables were extracted at pixel-level: the three RGB values and the temperature (*T_surf_*) that were subsequently scaled to the range 0 to 1.

An unsupervised pattern recognition method based on cluster analysis (k-means, 2 clusters) was applied to the normalised variables to separate the thermal image into two clusters of data. k-means cluster analysis (using Matlab R2024a) involved a two-phase iterative algorithm to minimise the sum of point-to-centroid distances summed over the 2 clusters [34]. After all pixels in the thermal image were categorised as being part of one of the two (cool or warm) areas, the proportion of the areas with lower temperatures in every thermal image was calculated as

(1) AcAw=ncnc+nw

where *n_c_* and *n_w_* are the number of pixels categorised by the cluster algorithm as being part of the cool and warm areas, respectively.

#### 2.4.2. Reflectance Spectra

The effect of solar light intensity fluctuations and tree leaves’ fluttering due to wind speed on canopy-level reflectance measurements has been highlighted by extensive past work on field spectroscopy [35]. In the next sections, the quality control criteria that were followed for the top-of-the-canopy reflectance spectra are detailed.

A Savitzky–Golay finite impulse response (FIR) smoothing filter (2nd polynomial order, 21 data points) was applied in the top-of-the-canopy spectra selected after the application of the quality control filters and the leaf-level measurements.

A thorough quality control of the measured canopy-level reflectance spectra was accomplished by checking the shortwave downward flux of solar radiation (*S_dw_*) during the reflectance measurements.

Based on experience gained during the measurement campaign, the following set of rules were selected and applied to the original set of 513 collected spectra:

(2) $$\frac{\sigma_{S_{dw}}}{\bar{S_{dw}}}<10\;{W\;m}^{-2},\;\bar{u}<5\;{m\;s}^{-1},\;\sigma_{u}<1\;{m\;s}^{-1}$$

$\bar{S_{dw}}$ is the mean and $\sigma_{S_{dw}}$ is the standard deviation of the shortwave downward flux of solar radiation over each measurement time period under examination. $\bar{u}$ and $\sigma_{u}$ are the mean and standard deviation of the mean half-hourly wind speed, respectively. The threshold for solar radiation ensured light was substantial and reasonably constant, and the threshold for wind speed variables ensured that the movement of the tree/tree foliage/crane was such that it was not adversely affecting the reflectance measurements. These thresholds were compared against records taken during the measurements. Spectrometric measurements taken with thermal images showing other than tree canopy surfaces were excluded from the database. After application of these filters, 304 spectra were left and were further processed, and results are presented in the main text of the manuscript and the Appendices. For the analysis discussed in the main text, 104 individual spectra, each one accompanied by thermal images taken on 13 August 2019, were further processed and discussed in the Section 3 of this manuscript. Raw data are presented in Figures S1 and S2.

### 2.5. Data Analysis

#### 2.5.1. Spectral Separability

The spectral separability between treatments was assessed by testing the statistical significance of reflectance spectral differences [6]. Spectral separability tests were performed on two groups of spectral reflectance measurements as follows: for every wavelength, a two-sided Wilcoxon rank sum test between reflectance spectra of the two groups (treatments) was applied; the null hypothesis tested (at α = 0.01) being that the two groups of measurements were taken from the same continuous distribution with no significant difference between their medians. Within each of the three wavelength bands (VIS, NIR, SWIR), the number of individual wavelengths for which the null hypothesis was rejected is reported in a percentage format and taken to represent spectral separability. The two treatments under consideration varied according to the occasion, and they were spectra between pairs of individual trees at leaf level (Section 3.2), spectra taken on two different heights at canopy level, and spectra corresponding to contrasting AcAw values (Section 3.3).

#### 2.5.2. Absorption Features

**Figure 3:**
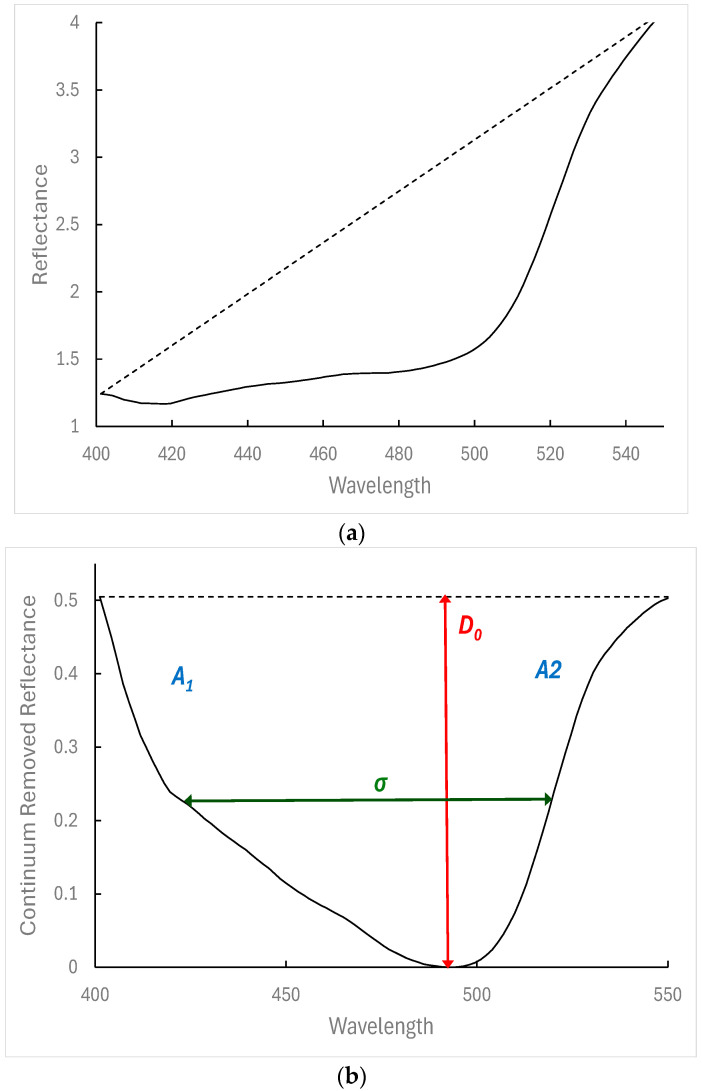
Example of continuum analysis for the absorption features between 400 nm and 550 nm for one of the canopy reflectance spectra shown in Figure 6: (**a**) measured reflectance spectrum between the two continuum endpoints of the feature (400 nm and 550 nm) and continuum line; (**b**) continuum-removed spectrum showing the main absorption features: absorption depth *(D*_0_*)*, width of the absorption center (full-width of feature at half-maximum absorption depth—*σ*), areas at the left and right of the absorption center (*A*_1_ and *A*_2_, respectively).

Absorption features were calculated for absorption bands commonly studied in vegetation optical studies and related to chlorophyll, carotenoids, water, dry matter, and nitrogen with the continuum removal method. The method aims to isolate and study individual absorption features of interest after removing other absorption features. Following [36], the absorption bands under examination were 400–550 nm, 550–750 nm (related to chlorophyll and chlorophyll and carotenoids Chlorophyll-1 and Chlorophyll-2, respectively), 920–1120 nm, 1072–1321 nm, 1370–1570 nm, 1670–1850 nm, 1870–2170 nm (related to water: water-1, water-2, water-3, water-4, and water-5, respectively), 1634–1783 nm, 2222–2378 nm (related to dry matter: dry matter-1 and dry matter-2, respectively), and 2010–2222 nm (related to nitrogen). A convex hull is applied over the selected spectrum band, and new normalised values between 0 and 1 (“continuum removed reflectance” values) were calculated after dividing the reflectance at a wavelength by the value of the hull at that wavelength [37]. For each one of the selected bands, the depth (*D*_0_), width (defined as the full wavelength width at half depth—*σ*), and area of the selected bands indicate the relevant absorption intensity (Figure 3); the asymmetry of the absorption band (*S*) defined as

(3) S=AleftAright

where *A_left_* (*A_right_*) is the absorption area to the left (right) of maximum absorption wavelength; *S* values greater (lower) than unity indicate an asymmetry skewed towards longer (shorter) wavelengths [38].

### 2.6. Numerical Experiments

The top of the canopy spectral reflectance R(λ) depends on a range of parameters involving biochemical and biophysical characteristics of the foliage and canopy, illumination conditions, the sensor’s viewing geometry, and soil conditions [39]. In particular:

- Biochemical characteristics of the tissue, i.e., leaf and non-photosynthetic vegetation (NPV: woody stem and standing litter, if available), affect their optical properties: leaf and NPV hemispherical reflectance and transmittance. Leaf optical properties are a function of the leaf water content, concentrations of biochemicals and leaf structure [40,41] such as chlorophyll a + b content (*C_ab_*, µg/cm^2^), carotenoids (carotenes + xanthophylls) content (*C_ar_*, µg/cm^2^), brown pigments content (*C_bp_*, in arbitrary units), equivalent water thickness (*C_w_*, cm), and dry matter content (*C_dm_*, g/cm^2^) and leaf structure parameter (*N*-the number of compact layers specifying the average number of air/cell walls interfaces within the mesophyll).
- Biophysical attributes (foliage clumping, leaf and stem area, and orientation) are mainly associated with the canopy architecture and play a critical role in describing the photon’s transport and interaction with the canopy [41,42]. They are represented by average leaf angle (*LIDF_a_*, degrees); Leaf Area Index (*LAI*, m^2^ m^−2^); and background (*r_soil_*, a unitless parameter that defines the percentage of grass and soil background).
- Sensor’s viewing geometry and illumination conditions, i.e., solar and sensor azimuth and elevation, directly affect spectrometric measurements [43], as well as the underlying surface’s (soil, grass) optical properties [39,41,44].

The biochemical and biophysical attributes of the warmer and cooler areas of the canopy for the given geometry of our measurements were investigated in a series of modelling runs with the well-known PROSPECT-5D [45] and PROSAIL-5D [46] radiative transfer numerical models: PROSPECT gives the reflectance and transmittance at leaf level and PROSAIL (that is, a fusion of PROSPECT and SAIL models) at canopy level. The codes used can be found at http://teledetection.ipgp.jussieu.fr/prosail/ (accessed on 1 January 2025). Model runs were conducted in both direct and inversion modes. In the inversion mode biophysical and biochemical properties of the leaf and canopy were extracted from the experimental measurements (Section 3.4). In the direct mode, the input parameters of the model are given, and the reflectance is calculated; this was used to test the sensitivity of the Photochemical Reflectance Index (*PRI*) to input Leaf Area Index (*LAI*) and carotenoid content values (Section 3.5).

## 3. Results and Discussion

### 3.1. Surface Temperatures: Separating Warmer from Cooler Canopy Areas

**Figure 4:**
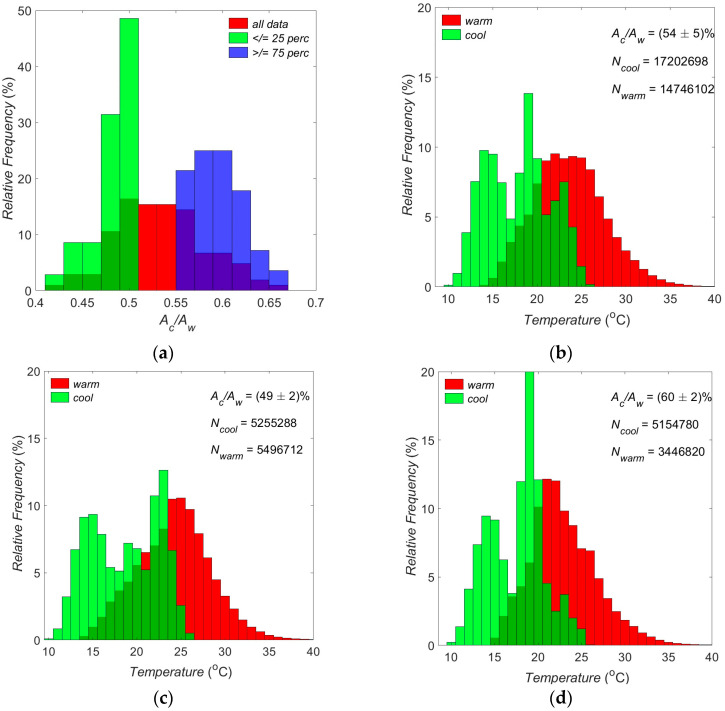
Histograms of relative frequency of the AcAw between cool and warm temperatures (**a**); surface temperature corresponding to all values (**b**); values in the first quantile (**c**); and values in the third quantile (**d**) of the AcAw parameter. Parameters listed in (**b**–**d**): cool to warm ratio: average (±standard deviation) of the AcAw parameter; *N_cool_* (*N_warm_*): number of pixels used to create the cool (warm) area histogram.

Results from the thermal imaging analysis introduced in Section 2.4.1 are presented in Figure 4. The AcAw parameter ranges between 0.42 and 0.65 with the mean value equal to 0.54 (54% of the total canopy surface captured in all 104 thermal images collected during 13 August 2019 corresponds to cooler areas and 46% to warmer areas). It is evident from Figure 4a that the entire dataset is slightly skewed towards cooler values (skewness = 0.20). The median temperature of the warmer canopy areas captured in all thermal images is $\tilde{T_{w}}=23.50\;{}^{\circ}C$ against $\tilde{T_{c}}=18.40\;{}^{\circ}C$ the median temperature of the cooler areas of this dataset (Figure 4b). Two sub-datasets were selected for further analysis:

- The first sub-dataset has 35 cases, and it corresponds to the first quartile of the AcAw distribution, i.e., it includes values in the range (0.42…0.51) with a median value of 0.49 (i.e., 49% of the total canopy surface captured in the 35 thermal images of the sub-dataset corresponds to cooler areas). The median surface temperature for this dataset is $\tilde{T}=22\;{}^{\circ}C$. The distribution is strongly negatively skewed (Figure 4a: skewness = −1.21), and the median temperature of the warmer (cooler) canopy areas is $\tilde{T_{w}}=24.40\;{}^{\circ}C$ ($\tilde{T_{c}}=18.90\;{}^{\circ}C$) (Figure 4c).
- The second sub-dataset consists of 28 cases (individual thermal images corresponding to valid reflectance spectra) from the third quartile of the AcAw distribution, i.e., it includes values in the range (0.57… 0.65), and it has a median value of 0.60 (i.e., 60% of the total canopy surface captured in the 28 thermal images of the sub-dataset, corresponds to cooler areas). The median surface temperature for this dataset is $\tilde{T}=19.5\;{}^{\circ}C$. The AcAw distribution is positively skewed (Figure 4a: skewness = 0.46), and the median temperature of the warmer (cooler) canopy areas is $\tilde{T_{w}}=22.50\;{}^{\circ}C$ ($\tilde{T_{c}}=18.40\;{}^{\circ}C$) (Figure 4d).

From the above discussion, it is clear that there is a mean difference of more than 2 °C between the two sub-datasets (corresponding to the first and third quartiles of the AcAw parameter), with the first quartile (AcAw≤25th percentile) being the warmer of the two. Therefore, an assumption is made here that thermal images, and hence spectral reflectance from the first sub-dataset (AcAw≤ 25th percentile), correspond to warmer areas of the canopy; spectral reflectance from the second sub-dataset (AcAw≥75th percentile) correspond to cooler areas of the canopy. The surface temperature distribution (evidenced by the mean temperature differences: mean value of the temperature difference between the warmer and cooler areas across all thermal images in each sub-category) appears similar for all sub-categories: a median value of ($\tilde{∆T_{all\;ratios}}=5.75\;{}^{\circ}C$ is found for all thermal images (Figure S3a); this value is 4.84 °C and 5.41 °C for the second and third datasets (corresponding to AcAw in the third and first quartiles, respectively; Figure S3a).

### 3.2. Leaf-Level Spectral Reflectance

**Figure 5:**
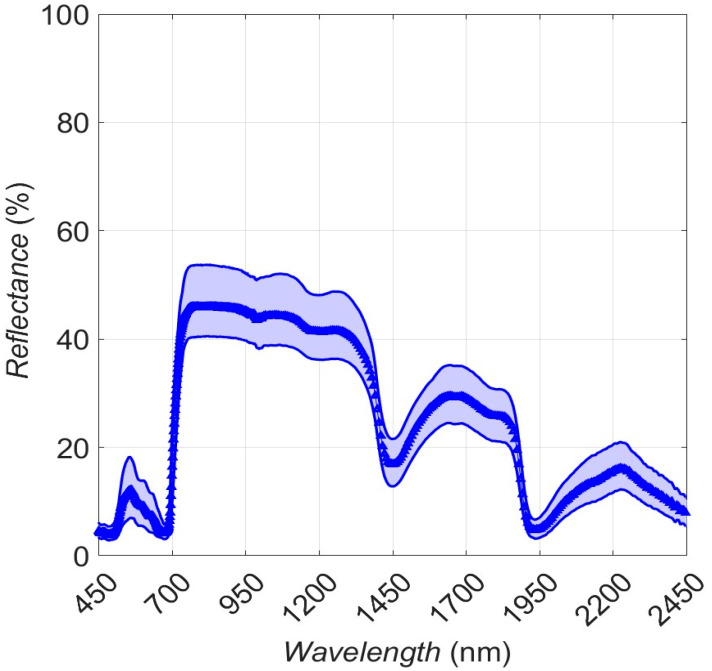
Composite plot of leaf-level reflectance spectra. Median reflectance is plotted as a solid thick line; the range between 5th and 95th percentiles as shaded areas.

In Figure 5, composite leaf-level reflectance spectra are presented (also in Figure S4 for the individual trees). The well-known major absorption features related to chlorophyll activity around 400–460 nm and 600–670 nm are clear [47]. The abrupt increase in the NIR (“red edge”) reflects the transition from the effects of strong chlorophyll absorption to a wavelength regime where the dominant event is the photon scattering by the internal leaf structure, i.e., at the air–cell interfaces within the mesophyll (“NIR plateau” [39,48]). The two weak local features located around 1000 nm and 1200 nm are characteristics of water absorption and are usually found in woody plants [39]. In the SWIR band, two major liquid water bands are evident and centered at 1400 nm and 1900 nm, respectively [49,50]. Signals in the SWIR reflectance spectra related to lignin or other carbon constituents are obscured because of the intense liquid water absorption bands.

The median leaf-level spectral reflectance values (Table S1) are 7.9%, 44.5%, and 17.1% for the VIS, NIR, and SWIR bands of the spectrum, respectively, in accordance with ranges reported elsewhere for broadleaf wooded vegetation [6,39]. Conversely, the mean reflectance variability (i.e., coefficient of variation) is higher for the VIS (21%) and lower for the NIR (3.4%) and SWIR (7%). This is in contrast with values reported for naturally grown trees [39], where reflectance variability was lower in VIS than NIR and SWIR, reflecting the stable optical properties of leaves due to the biochemically active pigments.

The spectral separability between all individual trees at the leaf level (Table S2) is generally rather low (average value 22%) across all three bands. Values range between 15% and 33%, with higher values detected for trees 6 and 5 (average spectra separability is 33% and 31%, as opposed to 25%, 17%, 15%, 21%, and 15% for trees 1, 2, 3, 4, and 7, respectively). In accordance with the mean variability, higher spectra separability is observed for the VIS bands (41%) and lower for the NIR (4%) and SWIR (22%) bands.

The absorption features discussed at the beginning of this section are further shown in Table 1 using the continuum removal method explained in Section 2.5.2. The most prominent absorption features indicating absorption intensity (*D*_0_, *σ*, and *A*) are related to chlorophyll and carotenoids (550–750 nm) and water (1870–2170 nm), followed by chlorophyll at 400–550 nm and water at 1370–1570 nm. All these absorption features are skewed to longer wavelengths, except water in 1870–2170 nm, which is skewed to shorter wavelengths.

### 3.3. Canopy-Level Spectral Reflectance

**Figure 6:**
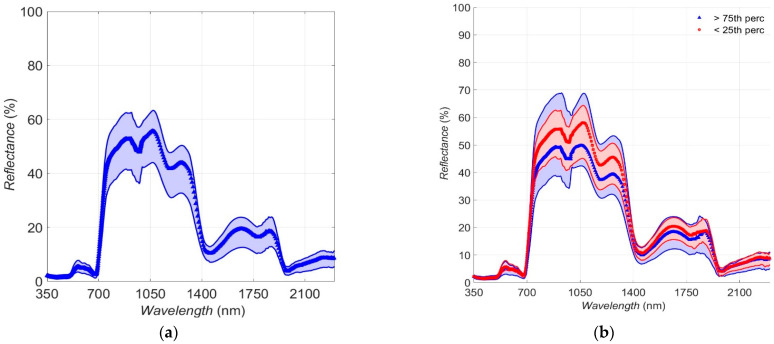
Composite plot of canopy-level reflectance spectra for (**a**) all measurements (104 individual spectra taken on 13 August 2019) and (**b**) measurements corresponding to the third (>75th) and first (<25th) quartiles of the AcAw ratio. Median reflectance is plotted as a solid thick line; the range between 5th and 95th percentiles as shaded areas.

The major features of the “chlorophyll well”, the “red edge”, and the “NIR plateau” discussed for the leaf-level spectra (Figure 4) can also be seen at the canopy-level spectra (Figure 6a,b). Compared to the leaf-level spectra, some significant differences are evident: mean reflectance values in the VIS and SWIR bands are lower in the canopy than the leaf level: 3% and 7.8% for the VIS and 15.5% and 17.5% for the SWIR, respectively. The opposite is observed for the NIR reflectance, with the canopy level values greater than the leaf level (51.5% and 45.2%, respectively). These results are in accordance with [6], where it was found that within broadleaf species, in comparison to leaf-scale spectra, branch-scale spectra had lower visible reflectance, higher NIR reflectance, and enhanced spectral contrast between NIR and SWIR. In the present study, enhanced spectral contrast between the NIR and SWIR bands in the canopy-level and leaf-level reflectance spectra is also observed (mean values: 36.1% and 27.7%, respectively). Another feature of interest is the apparent deepening of the two absorption bands in the NIR band of the spectrum (~1000 nm and 1200 nm) in comparison with the leaf-level spectra. These differences are also reflected in the spectral separability (Table 2) and absorption features (Table 3) between leaf and canopy level spectra.

The enhancement of the canopy reflectance and the deepening of the two absorption bands within the NIR band were also confirmed in the theoretical studies of References [39,51]. In the former, following a canopy spectral analogy, it was suggested that due to multiple scattering, the NIR biochemical signal can be significantly amplified at the canopy scale. In the latter, the variation of Leaf Area Index (*LAI*) was found to critically contribute to the modification of the biophysical signal in the NIR: an increase in the *LAI* (as evident in the broadleaf trees examined in this study) resulted in an enhancement of the biophysical signal and, therefore, of the NIR spectral reflectance. Whilst the overall NIR trend was toward increased scattering with increased leaf area, NIR plateau absorption features “lagged” behind the rest of the plateau due to enhanced water absorption as canopy biomass increased [39].

From Figure 6b, it is evident that the main difference between spectra corresponding to warmer and cooler areas (first and third percentile for the AcAw values) are detected mainly for the NIR and, to a lesser extent, for the SWIR band of the spectrum. These differences were further shown in the spectral separability results between all sub-sets at the canopy level and the ones at the leaf level (Table 2), where significant differences are detected between spectra corresponding to the warmer and cooler areas of the canopy (first and third quartiles of the AcAw parameter). Across the canopy-level spectra, the largest difference is observed for the NIR band, followed by differences in SWIR and finally in VIS bands. Interestingly, the cooler areas of the canopy are clearly distinguishable from the entire dataset (spectral separability is 44%, 100%, and 54.4% for the VIS, NIR, and SWIR bands, respectively), whilst the warmer areas (corresponding to the first AcAw quartile) have identical reflectance features (spectral separability is 0% for all spectral bands).

The basic absorption features found at the leaf level (Section 3.2) are also observed at canopy-level spectra (Tables S1–S3). Comparison of the absorption features across these datasets (Table 3) reveals significant differences between leaf and canopy levels across all wavebands; the main differences in absorption features between the warmer and cooler canopy areas (first and third quartile of the AcAw parameter), is observed for water absorption-related bands (centered at 975 nm, 1450 nm, and 1750 nm) as well as for the chlorophyll and carotenoid absorption bands. For the latter, it is not clear if changes are related to a weakening or strengthening of the absorption features: it appears that warmer areas are related to a decreased width of the absorption center (*σ*), but at the same time, the area increases. Absorption features related to chlorophyll:

Carotenoids (at 400–550 nm and 550–750 nm) in the warmer areas are skewed to longer wavelengths as compared to cooler areas (*S*-values are 2.48 and 2.78 compared to 2.28 and 2.55, respectively—Tables S3 and S6); the opposite is observed for the water-related absorption band (at 920–1120 nm) where cooler areas are skewed to longer wavelengths when compared against warmer areas (*S*-values are 1.99 and 0.72, respectively—Tables S3 and S4). Whilst in terms of reflectance features, as detected via spectral separability, the cooler areas of the canopy are distinguishable from the entire dataset, in terms of absorption features, it is the cooler areas that differ significantly from the entire dataset; thus, 58% of the absorption features differ significantly between the cooler areas and the entire dataset, against 7.5% for the warmer areas.

### 3.4. Inverse Modelling for Biochemical and Biophysical Attributes

In the following, a numerical modelling approach is performed, aiming to further explore the biochemical and biophysical characteristics of the studied canopy. Model inversions are performed using the leaf- and canopy-level datasets described in Section 3.2 and Section 3.3. The inverse modelling runs were conducted in three successive steps: in the first two steps, the range of the biochemical composition of the leaves and the biophysical attributes of the canopy, respectively, were identified; in the third step, the identified ranges were set as boundary values to improve the quality of the inversion.

#### 3.4.1. Range for Biochemical Composition Constituents at Leaf Level

In this first step, the range of the biochemical composition at the leaf level was identified by inverse modelling with the PROSPECT-5D model [52]. The range of the following six variables was estimated: leaf structure parameter (*N*—the number of compact layers specifying the average number of air/cell walls interfaces within the mesophyll), chlorophyll a + b content (*C_ab_*, µg cm^−2^), carotenoids (carotenes + xanthophylls) content (*C_ar_*, µg cm^−2^), brown pigments content (*C_bp_*, in arbitrary units), equivalent water thickness (*C_w_*, cm), and dry matter content (*C_dm_*, g cm^−2^). The initial and boundary values used in the inversion runs were *N*: 1.5, (1, 3.5); *C_ab_*: 50 µg cm^−2^, (0.0 µg cm^−2^, 100.0 µg cm^−2^); *C_ar_*: 10 µg cm^−2^, (0.0 µg cm^−2^, 30.0 µg cm^−2^), *C_bp_*: 0.1, (0, 1); *C_w_*: 0.01 cm, (0.00005 cm, 0.05000 cm); *C_dm_*: 0.01 g cm^−2^; (0.002 g cm^−2^, 0.020 g cm^−2^), respectively. Three runs were conducted, corresponding to the median, 1st percentile, and 99th percentile of the leaf-level reflectance values discussed in Section 3.2, and the results are shown in Table 4 and Figure S5. The *C_ab_*, C_w_, *C_dm_* values obtained from all runs (ranges: (28.45 µg cm^−2^ 44.38 µg cm^−2^), (0.10 cm, 0.12 cm), (0.007 g cm^−2^, 0.011 g cm^−2^)) are close to the median values reported in [53] which were compiled from 17 datasets: *C_ab_*: median ~40 µg cm^−2^ range (~0 µg cm^−2^ ~100 µg cm^−2^); *C_w_*: median ~0.01 cm, range: (~0.0001~0.05 cm); *C_dm_* (expressed as Leaf Mass Area): median ~0.01 g cm^−2^, range: (~0.00001~0.05 g cm^−2^). All acquired C_ar_ values are skewed to the upper quartile of the distributions found in [53], indicating the importance of carotenoid content in this dataset. The acquired values for *N* (1.19 2.28) and *C_bp_* (0.1) are well within the limits suggested within the code of the model ((1.0 3.5) and (0 1), respectively).

#### 3.4.2. Range for Biophysical Attributes at Canopy Level

In the second step, the range of the biophysical composition at the canopy level was identified by inverse modelling the PROSAIL-5D model [41]. The range of the following three variables was estimated: *LIDF_a_* (average leaf angle, degrees); *LAI* (Leaf Area Index); *r_soil_* (parameter that defines the percentage of grass and soil background parameters). Background surface (soil, grass) reflectance spectra are shown in Figure S6. The initial and boundary values used in the inversion runs were *LIDF_a_*: 45°, (−90° 90°); *LAI*: 4, (0.1 6.0); *r_soil_*: 0.5, (0 1) respectively. Solar zenith angle (*tts*) was set to 70° and observer zenith angle (*tto*) to 0°; hotspot (*q*) to 0.25. Nine runs were conducted: three sets corresponded to the median, 1st percentile, and 99th percentile of the canopy-level reflectance values discussed in Section 3.3; and for every set, there were three sets of runs corresponding to the 1st percentile, median, and 99th percentile of the *N*, *C_ab_*, *C_ar_*, *C_bp_*, *C_w_*, and *C_dm_* values from the previous section. The methodology for the inversion was identical to the one used in the previous section; the results are shown in Table 5 and Figure S7. The acquired *LAI* has a median value of 4 (*IQR* = 3); the respective values for *LIDF_a_* are 71° (*IQR* = 71°). *r_soil_* ranged between 0.9 and 0.99 in all cases, indicating a grass background.

#### 3.4.3. Biochemical and Biophysical Attributes of the Warmer and Cooler Areas of the Canopy

In this final step, the biochemical and biophysical attributes (*N*, *C_ab_*, *C_ar_*, *LIDF_a_*, *LAI*, *r_soil_*) of the warmer (AcAw≤25th percentile) and cooler (AcAw≥75th percentile) areas of the canopy were calculated. Taken from the previous runs, the initial and boundary values for all variables during these inversions were estimated as N: 1.47 and (1.19 2.28); *C_ab_*: 30 μg cm^−2^ and (28.45 μg cm^−2^ 44.4 μg cm^−2^); *C_ar_*: 15 μg cm^−2^ and (11.51 μg cm^−2^ 30 μg cm^−2^); *LIDF_a_*: 70° and (60° 80°); *LAI*: 3 and (0 6); *r_soil_*: 0.95 and (0.9 0.99). For these runs, following the results from steps 1 and 2, it was set *C_bp_* = 0.01; *C_w_* = 0.0102 cm; *C_dm_* = 0.0104 g cm^−2^; *tts* = 70°; *tto* = 0°; *q* = 0.25. The methodology followed was the same as in the previous runs. The reflectance datasets used for the inversions were the ones described in Section 3.3 (Figure 6), and the results are presented in Table 6.

The warmer areas of the canopy correspond to significantly higher *C_ar_* content (30 μg cm^−2^ compared to 11.69 μg cm^−2^ for the cooler canopy areas). This might be an indication of greater exposure to sunlight that results in higher temperatures and carotenoid content [36]; however, such an increase is not reflected at the chlorophyll concentrations, which were found similar for the two datasets (39.27 μg cm^−2^ and 36.63 μg cm^−2^ for the cooler and warmer parts of the canopy). Photoinhibition activity related to carotenoids might be another possible link between the observed increased temperature and carotenoid content: in [54], it was found that following overexposure to sunlight in the vine *Smilax australis*, the chlorophyll/carotenoid ratio decreased. Even though photoinhibition occurs at several scales (p. 46 in [36]), further research is needed to conclude whether the phenomena studied here are of relevant temporal scales.

*LAI* is higher for the cooler parts of the canopy (4.26 and 2.74 for the cooler and warmer parts of the canopy, respectively), which might suggest a dependence on the relative positioning within the canopy, e.g., the warmer areas might be located at the sides and the cooler areas at the center of the canopy. It is important to note that *LAI* also depends on the vertical structure of the canopy [55].

### 3.5. Photochemical Reflectance Index (PRI)

*PRI* is commonly used as an indicator of the plant’s photosynthetic efficiency and is defined as

(4) $$PRI=\frac{R_{530}-R_{570}}{R_{530}+R_{570}}$$

**Figure 7:**
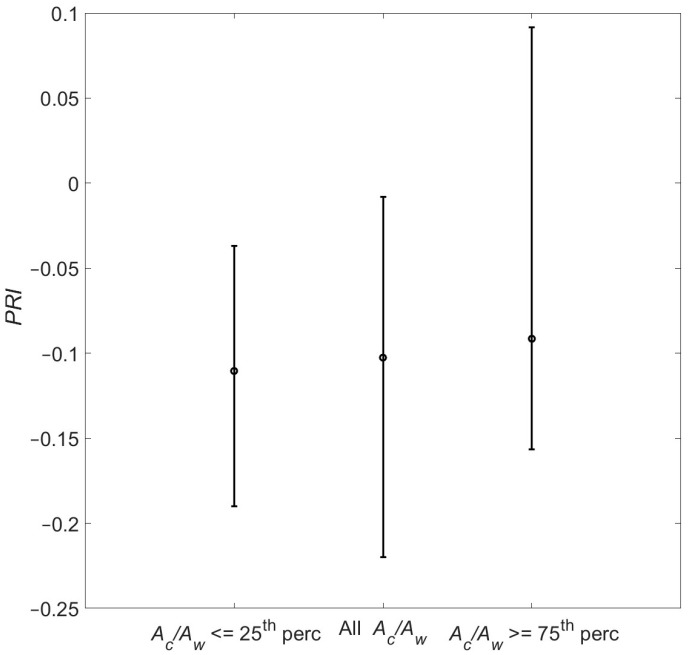
*PRI* obtained from the different datasets. Bullets correspond to median values and error bars to 1st and 99th percentiles.

*PRI* was calculated for the canopy spectral reflectance datasets described in Section 3.3: one value of the index was calculated for each individual canopy reflectance spectrum, and then this was repeated for all spectra across all three datasets (AcAw values, AcAw≤25th percentile, AcAw≥75th percentile). *PRI* has greater values in the AcAw≤25th percentile dataset and lower values in the AcAw≥75th percentile dataset, and the value for all AcAw lies in between (Figure 7).

Non-parametric statistical tests (Komlogorov–Smirnov two-tailed test with a null hypothesis that the datasets are drawn from the same distribution; Mann–Whitney test with a null hypothesis that the two groups of measurements were taken from the same continuous distribution with no significant difference between their medians) were conducted between each pair of datasets, and results are presented in Table 7. It is evident that whilst similar *PRI* values are obtained from all AcAw and the AcAw≤25th percentile datasets (both statistical tests accept the null hypothesis), they differ between all AcAw and the AcAw≥75th percentile. It is worth noting that these results are in accord with the spectral separability results, where most differences (Table 2) were found between all AcAw and the AcAw≥75th percentile datasets. Different *PRI* values are obtained from the AcAw≤25th percentile and AcAw≥75th percentile datasets (α ≤ 0.01, null hypothesis rejected), and these differences are further explored below.

**Figure 8:**
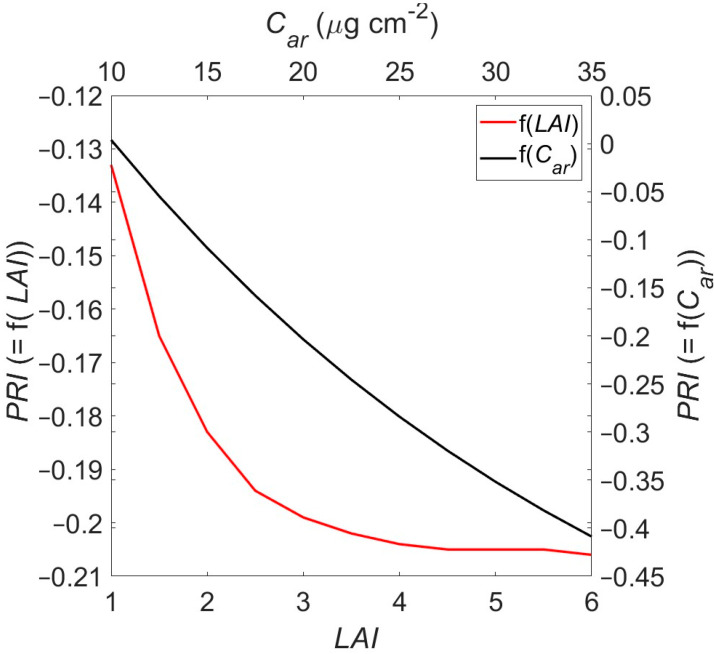
Sensitivity of *PRI* to *LAI* and carotenoid (*C_ab_*) content variability. Application to canopy-simulated reflectance using PROSAIL-5D model.

From the inversion runs discussed in Section 3.4, it was evident that the main differences between the two sub-datasets (AcAw≤25th percentile and AcAw≥75th percentile) were in terms of carotenoid content, C_ar_, and LAI. In order to qualitatively explore the sensitivity of the *PRI* on *C_ar_* and *LAI*, forward runs with the PROSAIL-5D model were conducted; in those runs, the input values were the same as the ones found from the inverse modelling runs, i.e., *N* = 2.28, *C_ab_* = 38 µg cm^−2^, *C_bp_* = 0.01; *C_w_* = 0.0102 cm; *C_dm_* = 0.0104 g cm^−2^; *LIDF_a_* = 70°, *r_soil_* = 0.99, *tts* = 70°; *tto* = 0°; *q* = 0.25, whilst *C_ar_* and *LAI* values ranged between (10 µg cm^−2^ 35 µg cm^−2^) and (0 6), respectively. Results are presented in Figure 8.

*PRI* decreases as *C_ar_* (*LAI*) increases, and *LAI* (*C_ar_*) is constant. These results are consistent with Garrity et al. (2011), who found that *PRI* increases with carotenoid content as *C_ab_* values are kept constant (note that in their case, *PRI* was calculated considering the $R_{570}-R_{530}$ difference instead of $R_{530}-R_{570}$ used here). Also, in [56], it was suggested that *PRI* decreases as *LAI* increases. It is important to note, however, that the slope is sharper for *C_ar_* than for *LAI* in the range of *C_ar_* and *LAI* values found here ((10 30) and (2 4), respectively). It is, therefore, suggested that the lower *PRI* values for the AcAw≤25th percentile dataset seen in Figure 7 reflects the significantly higher carotenoid content found there; should the *LAI* difference be smaller (or reversed, with cooler areas having smaller *LAI* values), it would be expected that *PRI* would be even lower for the warmer areas of the canopy. To put it another way, the antagonistic effect of *LAI* and carotenoid content might be responsible for the very low, albeit statistically significant, *PRI* change seen here.

## 4. Conclusions

Differences in the biophysical and biochemical properties between the warmer and cooler areas of tree canopy with an experimental layout for characterizing the top-of-the-canopy thermal and spectral characteristics at the fine scale have been presented here. The method consisted of a combination of measurements taken with a spectrometer and a thermal camera mounted on a 7.3 m portable crane. Spectral reflectance was measured above the canopy of a cluster of trees, and thermal images were used to characterise illumination conditions and basic biophysical attributes associated with radiometric measurements of the canopy.

A new parameter AcAw that accounts for the proportion of the areas with lower temperatures in every thermal image was introduced, and two sub-datasets were selected with spectra corresponding to the warmer and cooler areas of the canopy with a mean surface temperature difference of ~2 °C. It was found that the two datasets differ statistically in terms of reflectance in the VIS, NIR, and SWIR bands and absorption features related to the chlorophyll, carotenoid, and water absorption bands. These differences were reflected in the Photochemical Reflectance Index, with *PRI* values decreasing as surface temperature increased.

Direct and inverse mode runs with the PROSPECT-5D and PROSAIL-5D models indicated that the observed differences were related to higher carotenoid content and lower *LAI* values of the warmer canopy areas. As these values have an antagonistic effect on *PRI*, it was apparent that the effect of the surface canopy temperature on *PRI* is significant.

As synergies and fusion between VISWIR and TIR data aiming to retrieve additional information and provide more accurate predictions are becoming more used in the areas of urban management [12], terrestrial ecology [57], geology [58], and agriculture [59], our results in the fine scaleshow that different biophysical and biochemical processes dominate in adjacent areas of the same canopy, suggest that when these synergies are applied at a large scale, they should be used with caution.

Furthermore, with the resolution of data products from air- and space-borne instruments increasingly improving (and fine-scale measurements becoming available), the results of this study indicate the potential of leveraging the synergy between thermal and spectral measurements for the purpose of more accurately assessing the complex biochemical and biophysical characteristics of measured vegetation canopies. The AcAw parameter introduced in the current study was used to separate the warmer from the cooler areas of the tree canopy on a small scale; this can be applied to data obtained from air- or space-borne sensors in order to disentangle the components of spectral reflectance at the crown scale. This approach parallels the approach found in [60], where lidars are used in a way to obtain shade-canopy masking, where errors caused by intra-canopy, inter-canopy, and canopy-to-ground shade were removed altogether from the analysis. As the AcAw parameter is an indicator of the shadowed and sunlit areas of the trees, it can be used in the context of data obtained from air- or space-borne instruments to identify pixels on a tree crown that are sunlit vegetation and, thus, are most free from NPV, shadow, or background effects.

Another area in this study that could prove useful is the study of green infrastructure in the urban environment, where the spatial heterogeneity of remotely sensed fields on a fine scale obfuscates the accurate interpretation of spectral signatures. Here, the AcAw parameter could be used as an indicator of the warmer underlying urban surfaces in a pixel with cooler greenery; this extra information, when fused with spectral measurements, could provide a promising method for disentangling the combined signal components in the urban areas, especially as the resolution of spaceborne instrument products rapidly improves.

## Figures and Tables

**Table 1:** Statistics for absorption feature statistics (absorption depth—*D_0_*, width of the absorption center—*σ*, area and asymmetry of the absorption band (*A* and *S*, respectively) at leaf level for the following wavebands: 400–550 nm, 550–750 nm (related to chlorophyll: Chlorophyll-1 and Chlorophyll-2, respectively), 920–1120 nm, 1072–1321 nm, 1370–1570 nm, 1670–1850 nm, 1870–2170 nm (related to water: water-1, water-2, water-3, water-4, and water-5, respectively), 1634–1783 nm, 2222–2378 nm (related to dry matter: dry matter-1 and dry matter-2, respectively), and 2010–2222 nm (related to nitrogen).

	*D*_0_			*σ*			*A*			*S*		
	Median	95th Percentile	5th Percentile	Median	95th Percentile	5th Percentile	Median	95th Percentile	5th Percentile	Median	95th Percentile	5th Percentile
Chlorophyll-1	0.69	0.75	0.60	114.40	118.80	105.10	71.52	79.56	63.27	1.71	2.57	0.67
Chlorophyll-2	0.88	0.90	0.85	123.90	138.15	88.18	99.14	114.68	78.69	2.78	3.35	2.12
water-1	0.03	0.04	0.03	38.60	53.90	22.70	1.84	2.42	1.35	0.75	1.30	0.47
water-2	0.03	0.04	0.02	83.50	89.90	83.50	2.74	3.54	2.18	0.67	0.89	0.52
water-3	0.46	0.54	0.40	110.70	116.90	110.70	51.93	61.86	44.22	0.54	0.68	0.53
dry matter-1	0.01	0.01	0.00	66.90	121.40	12.20	0.26	0.39	0.15	1.13	8.27	0.13
water-4	0.03	0.04	0.02	60.90	73.10	54.80	1.93	2.57	1.60	1.18	2.09	0.72
water-5	0.74	0.78	0.70	148.60	161.00	142.40	117.81	131.17	106.71	0.37	0.38	0.31
nitrogen	0.02	0.03	0.01	37.00	67.93	12.30	0.53	1.16	0.19	1.61	3.73	0.56
dry matter-2	0.05	0.07	0.04	59.70	95.90	11.90	2.89	4.26	2.11	1.74	11.07	0.59

**Table 2:** Spectral separability between leaf-level reflectance spectra and spectra corresponding to all, first (≤25th percentile), and third (≥75thpercentile) quartitle values of the AcAw for three wavebands: visible (VIS: 450–700 nm), near-infrared (NIR: 750–1400 nm), and shortwave infrared (SWIR: 1400–2300 nm). The spectral separability reports the percentage of total bands that were significantly different (*a* = 0.01), as shown from two-sided Wilcoxon rank sum tests.

Leaf/Canopy Level	Wavebands	All AcAw	AcAw≥75th Percentile	AcAw≤25th Percentile
AcAw≥75th percentile	VIS	44	-	
	NIR	100	-	
	SWIR	54.4	-	
AcAw≤25th percentile	VIS	0	74.9	-
	NIR	0	100	-
	SWIR	0	89.1	-
Leaf level	VIS	100	100	100
	NIR	76.5	43.3	86.2
	SWIR	99.3	98	98.6

**Table 3:** Statistics for differences in absorption features between leaf level and the first (AcAw≤ 25th percentile), third (AcAw≥75th percentile) and all AcAw quartiles at canopy level reflectance. All other variables are same as in Table 1. Statistics are reported in terms of two-sided Wilcoxon rank sum test *p*-values; the null hypothesis tested is that the two groups were taken from the same continuous distribution with no significant difference between their medians. Values in bold indicate rejection of the null hypothesis (indicating differences between the two groups) at significance level α = 0.05.

		All AcAw				AcAw ≥ 75th Percentile				AcAw ≤ 25th Percentile			
		*D*_0_	*σ*	*A*	*S*	*D*_0_	*σ*	*A*	*S*	*D*_0_	*σ*	*A*	*S*
AcAw ≥ 75th percentile	Chl-1	0.51	0.05	0.49	0.12	-	-	-	-				
	Chl-2	0.35	**0**	**0.01**	**0**	-	-	-	-				
	wat. 1	0.04	0.22	0.25	0.03	-	-	-	-				
	wat. 2	0.40	0.44	0.56	0.20	-	-	-	-				
	wat. 3	0.56	0.27	0.46	0.44	-	-	-	-				
	d.m.-1	0.40	0.05	0.93	0.77	-	-	-	-				
	wat. 4	0.34	0.83	0.32	0.92	-	-	-	-				
	wat. 5	0.59	0.96	0.70	0.88	-	-	-	-				
	Nitr.	0.05	0.96	0.06	0.64	-	-	-	-				
	d.m.2	0.77	0.61	0.52	0.53	-	-	-	-				
AcAw ≤ 25th percentile	Chl-1	0.61	**0**	**0**	**0**	0.42	**0**	**0.03**	**0**	-	-	-	-
	Chl-2	0.49	0.35	0.73	**0**	0.19	**0**	**0.01**	**0**	-	-	-	-
	wat. 1	**0.02**	**0**	**0**	**0**	**0**	**0**	**0.04**	**0**	-	-	-	-
	wat. 2	0.63	**0**	0.57	0.70	0.86	**0.02**	0.88	0.12	-	-	-	-
	wat. 3	**0**	**0**	**0**	0.69	**0**	**0**	**0**	0.84	-	-	-	-
	d.m.-1	**0**	**0**	**0**	0.14	**0**	0.10	**0**	0.34	-	-	-	-
	wat. 4	**0**	**0**	**0**	0.09	**0**	**0.02**	**0**	0.18	-	-	-	-
	wat. 5	0.29	**0**	**0**	0.04	0.20	**0**	**0**	0.19	-	-	-	-
	Nitr.	**0.03**	0.17	0.51	**0.03**	0.98	0.30	0.34	0.06	-	-	-	-
	d.m.2	**0**	**0**	**0**	0.22	**0**	**0**	**0**	0.67	-	-	-	-
Leaf	Chl-1	**0**	**0**	**0**	**0**	**0**	**0**	**0**	**0**	**0**	**0**	**0**	**0**
	Chl-2	**0**	**0**	**0**	**0**	**0**	**0**	**0**	**0**	**0**	**0**	**0**	0.66
	wat. 1	**0**	**0**	**0**	**0**	**0**	**0**	**0**	**0**	**0**	**0**	**0**	0.42
	wat. 2	**0**	**0**	**0**	**0**	**0**	**0**	**0**	**0**	**0**	**0**	**0**	**0**
	wat. 3	**0**	**0**	**0**	**0**	**0**	**0**	**0**	**0**	**0**	**0**	**0**	**0**
	d.m.-1	**0**	**0**	**0**	**0.03**	**0**	**0**	**0**	0.16	**0**	**0**	**0**	**0**
	wat. 4	**0**	**0**	**0**	0.24	**0**	**0**	**0**	0.53	**0**	**0**	**0**	**0**
	wat. 5	**0.03**	**0**	0.06	**0**	0.05	**0**	0.09	0.02	0.63	**0.01**	**0**	**0**
	Nitr.	**0**	**0**	**0**	**0**	**0**	**0**	**0**	**0**	**0**	**0.02**	**0**	**0**
	d.m.2	**0**	**0**	**0**	**0**	**0**	**0**	**0**	0.28	**0**	**0**	**0**	0.29

**Table 4:** Range of the biochemical composition of the leaves obtained during the PROSPECT-5B inversion (Step 1 of the model inversion). *N*: number of compact layers specifying the average number of air/cell walls interfaces within the mesophyll, *C_ab_*: chlorophyll a + b content *C_ar_*: carotenoids (carotenes + xanthophylls) content; *C_bp_*: brown pigments content, *C_w_*: equivalent water thickness; and *C_dm_*: dry matter content.

	*N*	*C_ab_* (µg cm^−2^)	*C_ar_* (µg cm^−2^)	*C_bp_* (Arbitrary Units)	*C_w_* (cm)	*C_dm_* (g cm^−2^)
1st percentile	1.19	44.38	29.99	0.010	0.012	0.011
median	1.47	28.92	12.95	0.010	0.010	0.007
99th percentile	2.28	28.45	11.51	0.010	0.010	0.010

**Table 5:** Range of the biophysical attributes at the canopy level obtained during the PROSAIIL-5D inversion (Step 2 of the model inversion). *N*: number of compact layers specifying the average number of air/cell walls interfaces within the mesophyll, *C_ab_*: chlorophyll a + b content *C_ar_*: carotenoids (carotenes + xanthophylls) content; *C_bp_*: brown pigments content, *C_w_*: equivalent water thickness; and *C_dm_*: dry matter content. *LIDF_a_*: average leaf angle, degrees); *LAI*: Leaf Area Index; *r_soil_*: parameter that defines the percentage of grass and soil background parameters. Perc: percentile.

		All AcAw			AcAw ≤ 25th Percentile			AcAw≥ 75th Percentile		
		1st Perc	Median	99th Perc	1st Perc	Median	99th Perc	1st Perc	Median	99th Perc
*N* = 1.47; *C_ab_* = 28.92; *C_ar_* = 12.95; *C_bp_* = 0.01; *C_w_* = 0.01; *C_dm_* = 0.01	*LIDFa*	78	65	46	77	63	75	78	69	71
	*LAI*	5.7	4.7	4.0	4.8	4.3	2.3	5.6	5.8	2.5
	*r_soil_*	0.99	0.99	0.99	0.99	0.99	0.95	0.99	0.99	0.99
	Relative error	13.98	13.43	15.01	12.48	13.32	13.73	14.61	14.56	13.90
*N* = 1.19; *C_ab_* = 44.38; *C_ar_* = 30; *C_bp_* = 0.01; *C_w_* = 0.012257; *C_dm_* = 0.01058	*LIDFa*	74	75	72	84	75	71	75	60	65
	*LAI*	4.9	2.5	2.1	3.0	2.4	2.0	4.8	5.6	2.1
	*r_soil_*	0.99	0.93	0.99	0.95	0.96	0.97	0.99	0.99	0.99
	Relative error	14.06	13.41	14.38	12.84	13.21	13.52	14.50	14.96	13.81
*N* = 2.28; *C_ab_* = 28.453; *C_ar_* = 11.517; *C_bp_* = 0.01; *C_w_* = 0.0102; *C_dm_* = 0.0104	*LIDFa*	79	69	65	81	70	67	80	70	62
	*LAI*	4.4	3.3	2.4	3.6	3.0	2.4	4.4	4.2	2.5
	*r_soil_*	0.99	0.99	0.99	0.99	0.99	0.99	0.99	0.99	0.99
	Relative error	13.91	13.23	14.48	12.57	13.19	13.47	14.53	14.37	14.56

**Table 6:** Results from the PROSAIL-5D inversions for the warmer and cooler areas of the canopy. *N*: number of compact layers specifying the average number of air/cell walls interfaces within the mesophyll, *C_ab_*: chlorophyll a + b content *C_ar_*: carotenoids (carotenes + xanthophylls) content; *LIDF_a_*: average leaf angle, degrees); *LAI*: Leaf Area Index; *r_soil_*: parameter that defines the percentage of grass and soil background parameters.

	*N*	*C_ab_*	*C_ar_*	*LIDFa*	*LAI*	*r_soil_*
AcAw > 75 percentile	2.28	39.27	11.69	69.72	4.26	0.99
AcAw < 25 percentile	2.28	36.63	30.00	73.72	2.74	0.99

**Table 7:** Statistical inference test results of PRI between the warmer and cooler areas of the canopy. KS: Kolmogorov–Smirnov test; MW: Mann–Whitney test.

Datasets	AcAw ≤ 25th Percentile		All AcAw	
	KS	MW	KS	MW
All AcAw	0.129	0.035		
AcAw ≥ 75th percentile	0.000	0.000	0.007	0.001

**Figure A1:**
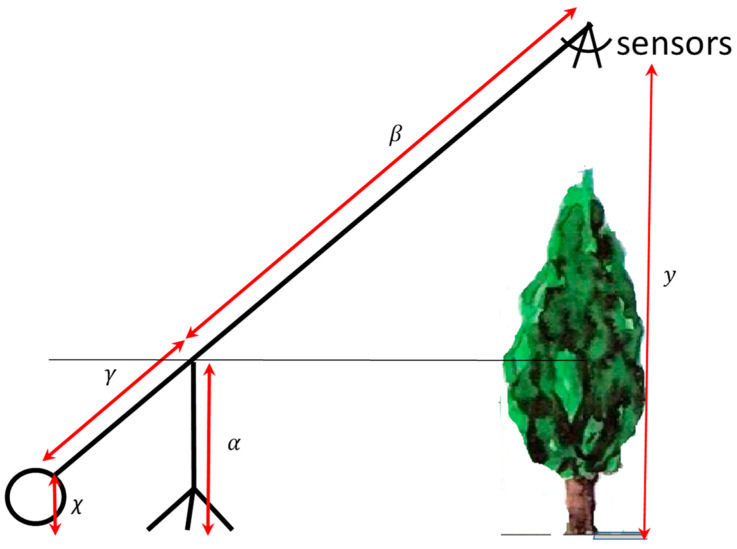
Schematic representation of the jib crane.

**Figure A2:**
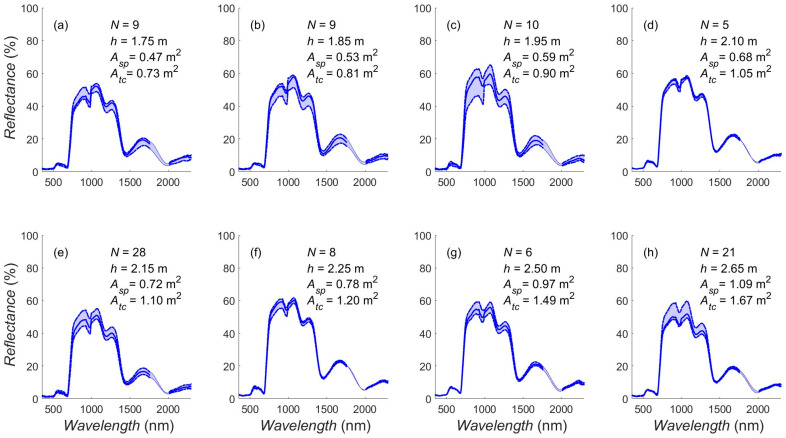
Composite plots of top of the canopy spectral reflectance measurements (Stage i) for measurements taken at 1.75 m (**a**), 1.85 m (**b**), 1.95 m (**c**), 2.10 m (**d**), 2.15 m (**e**), 2.25 m (**f**), 2.50 m (**g**), 2.65 m (**h**) above the mean canopy height. Median reflectance is plotted as a solid thick line; interquartile range (25th–75th percentiles) as dotted lines surrounding shaded areas. Parameters listed: *N*: number of spectra measured, *A*: source area for the measurements taken with the spectrometer (*A_sp_*) and the thermal camera (*A_tc_*). *h*: height of the sensors above mean canopy level.

## Data Availability

Data used in the paper are available at the University of Reading Data Repository (URL to follow).

## Supplementary Materials

The following supporting information can be downloaded at: https://www.mdpi.com/article/10.3390/s25030962/s1, Table S1: Leaf-level reflectance median values for three wavebands: visible (VIS: 300 nm–700 nm), near infrared (NIR: 750 nm–1400 nm) and shortwave infrared (SWIR: 1400 nm -2400 nm), Table S2: Spectral separability at leaf-level between all trees for three wavebands: visible (VIS: 300 nm–700 nm), near infrared (NIR: 750 nm–1400 nm) and shortwave infrared (SWIR: 1400 nm–2400 nm). The spectral separability reports the percentage of total bands that were significantly different (*a*= 0.01) as shown from two-sided Wilcoxon rank sum tests, Table S3: As in Table 1 but for the canopy reflectance (all measurements), Table S4: As in Table 1, but for spectra corresponding to the third percentile of the AcAw parameter, Table S5: As in Table 1, but for spectra corresponding to the first percentile of the AcAw parameter. Table S6: Details of spectrometric measurements taken during the first stage of the experimental period (13/8/2019) at the cluster of trees, Table S7: Details of spectrometric measurements taken during the second stage of the experimental period (17-20/9/2019) at the cluster of trees, Table S8. Correlation coefficients between top of the canopy reflectance in the VIS, NIR and SWIR wavelength bands, and controlling variables during Stage (ii) of measurements, Figure S1: Raw spectral reflectance measurements at canopy scale used in this study, Figure S2: Raw surface temperature measurements at canopy scale used in this study, Figure S3: Histograms of relative frequency of the median (a), the lower (1st) (b) and higher (99th) (c) percentiles of the surface temperature difference between the cool and warm parts of the canopy for all values and values in the first and third quartile of the AcAw parameter, Figure S4: Composite plots of leaf-level reflectance spectra for Tree 1-7 (a–g respectively). Median reflectance is plotted as a solid thick line; interquartile range (25th–75th percentiles) as dotted lines surrounding shaded areas, Figure S5: Comparison between leaf-level modeled (PROSPECT-5B) and measured reflectance spectra for the median (a) 1st and 99th percentile values of spectral reflectances measured at leaf-level. N: number of compact layers specifying the average number of air/cell walls interfaces within the mesophyll, Cab: chlorophyll a+b content Car: carotenoids (carotenes + xanthophylls) content; Cbp: brown pigments content, Cw: equivalent water thickness; and Cdm: dry matter content. RMSE: Root Mean Square Error; RE: relative error (RMSE/mean), Figure S6: Measured soil (a) and grass (b) reflectance spectra, Figure S7: Comparison between leaf-level modeled (PROSPECT-5) and measured reflectance spectra for the median (a) 1st and 99th percentile values of spectral reflectances. N: number of compact layers specifying the average number of air/cell walls interfaces within the mesophyll, Cab: chlorophyll a + b content Car: carotenoids (carotenes + xanthophylls) content; Cbp: brown pigments content, Cw: equivalent water thickness; and Cdm: dry matter content. RMSE: Root Mean Square Error; RE: relative error (RMSE/mean), Figure S8: Scatter plots between sorted and binned values of the proportion of areas with lower temperatures captured in thermal images (AcAw) and mean reflectance at the VIS (a), NIR (b) and SWIR (c) wavelength bands. The data were sorted according to AcAw ascending order and then binned in clusters of 10 points. The data shown are the mean values of AcAw and $\bar{\;R_{VIS}}$, $\bar{\;R_{NIR}}$ and $\bar{\;R_{SWIR}}$ within each one of these bins, Figure S9: Composite plot of top of the canopy spectral reflectance measurements (Stage ii). Median reflectance is plotted as a solid thick line; interquartile range (25th–75th percentiles) as shaded areas. h: height of the sensors above mean canopy level, Figure S10: Scatter plot of binned mean reflectance at the NIR wavelength band and the regressed variable RegR. The presented data are the mean values within bins of ten datapoints of measurements. The blue line is the 1:1 line.

## Appendix A. Height of Measurements Calculation

The height of the sensors, *y*, was calculated by measuring the height, *x*, at the lower end of the crane using the following formula:

$$y=\alpha +\frac{\beta }{\gamma }\left(a-x\right)$$

Distances *α*, *β*, and *γ* were measured as 1.75 m, 5.40 m, and 1.45 m, respectively.

## Appendix B. Ancillary Measurements

An IRGASON-integrated CO_2_ and H_2_O Open-Path Gas Analyzer and 3-D Sonic Anemometer system (Campbell Scientific, Logan, UT, USA) measured momentum and convective energy (sensible and latent heat) fluxes with the eddy covariance method. Conventional meteorological measurements were also obtained with the system (wind speed and temperature). The system was mounted on a meteorological mast 50 m away from the tree cluster at 5.3 m above ground level (a.g.l.). The sampling rate was set to 10 Hz, and mean values were output every 30 min. A SN-500 Apogee Net Radiometer 5M (Apogee Instruments, Logan, UT, USA) was mounted on the mast at 5 m a.g.l. measuring the short- and long-wave incoming and outgoing components of the net radiation and output values every 10 secs. The mast was installed on 16 July and stayed until the end of the experiment.

Air temperature and relative humidity were measured with 5 Tinytag dataloggers (Gemini Data Loggers, Chichester, UK) attached to the trees at 1.2 m a.g.l. outputting mean values every minute. Decagon 5TM soil temperature and water content and Teros 21 volumetric water potential sensors (Decagon Devices, Inc., Pullman, WA, USA) were installed at 10 cm in containers’ soil. Decagon and Tinytag sensors were attached to the respective trees (Figure 2) on 3 June and 11 June, respectively, and followed the trees in different arrangements (Section 3.3). A Handy PEA+ continuous excitation chlorophyll fluorimeter (Hansatech Instruments Ltd., Norfolk, UK) was used to record tree condition (stress) on an almost weekly basis.

## Appendix C. Testing the Method: Canopy Reflectance Measurements Conducted on a Cluster of Trees

In this section, the canopy reflectance measurements conducted on a cluster of trees will be used in order to test the robustness of the method against the height of measurements. As described in Section 2.3, measurements have been conducted in two stages:

(i) Measurements were taken from multiple heights: a total of 36 sets of spectrometric measurements (138 spectra and thermal images) at nine different heights were taken between 10:50 and 14:17 on 13 August (Table S6). During this period solar elevation was reasonably stable, changed by 17% ranging between 44° and 53°, and the solar azimuth ranged between 130° and 207°. During this period, moderate westerly winds prevailed (half-hourly wind speed measured at 5.3 m a.g.l averaged at 2.7 m s^−1^, standard deviation: 0.4 m s^−1^). Scattered clouds developed in the last part of the measurements; the shortwave incoming solar radiation averaged at 776 W m^−2^ (standard deviation 251 W m^−2^).
(ii) During the period 17–20 September, 57 sets (375 individual spectra and thermal images) of measurements were collected with the sensors placed at two heights only: 5.45 m and 5.85 m a.g.l. During the measurements, the solar elevation changed by 15% (ranged between 34° and 40°), and the solar azimuth ranged between 174° and 220°. Following the procedure described previously, cycles of measurements were alternating between the two heights across the four days (Table S7). During this period, the prevailing wind gradually changed from N to NE and finally to E. The half-hourly wind speed measured at 5.3 m a.g.l. during the measurements averaged at 2.6 m s^−1^ (standard deviation 1.4 m s^−1^) with higher wind speeds prevailing during the 20/9 (4.75 m s^−1^ ± 0.6 m s^−1^ mean and standard deviation values, respectively). The sky was cloudless during this period, and the shortwave incoming solar radiation averaged at 605 W m^−2^ (standard deviation 50 W m^−2^).

In the following, results from Stage (i) (measurements taken at multiple heights) are described in Section (a), and results from Stage (ii) (measurements taken at two heights) are described in Section (b) of this Appendix.

(a) Measurements taken at multiple heights: exploring the controlling parameters.

The top of the canopy reflectance for different heights (1.75 m, 1.85 m, 1.95 m, 2.10 m, 2.15 m, 2.25 m, 2.50 m, 2.65 m above the mean canopy height) is presented in Figure A2 and Table A1. The major absorption features discussed for the leaf-level spectra (Figure S4) can also be seen in the canopy-level spectra (Figure A2).

The median canopy-level spectral reflectance values across all heights (Table A1) range between 2.0% and 3.5% for the VIS, 44.0% and 55.1% for the NIR, and 10.3% and 14.3% for the SWIR bands of the spectrum, respectively (coefficient of variation are 18.8%, 7.3% and 11.5% for VIS, NIR, and SWIR, respectively).

In Table A1, the control parameters that influence reflectance are also reported. Some of these control parameters refer to the influence of the meteorological conditions discussed in Section 2.4.2: the mean and mean coefficient of variation for the shortwave downward flux of solar radiation (σSdw Sdw¯)) and the mean and standard deviation of the half-hourly wind speed ($\bar{U}$ and $\sigma_{U}$) during each cycle of measurements (i.e., every height).

From Figure A2, it can be seen that as the sensors are placed higher above the canopy (h has higher values), the sensors’ FOV captures larger areas, and the parameters *A_sp_* and *A_tc_* (the source area of the sensors) increase as well. Therefore, during the comparison of spectra obtained from different heights, caution should be taken in order to address differences related to illumination conditions and the biophysical attributes of the areas, reflecting differences in the areas captured within the FOV of the sensors.

In Table A1, the proportion of areas with lower temperatures captured in thermal images (AcAw) and also statistical values of temperatures in the cooler ($\bar{T_{c}}$, $\min T_{c}$, $\max T_{c}$) and warmer ($\bar{T_{w}}$, $\min T_{w}$, $\max T_{w}$) areas of the thermal images are presented. These control parameters are related mainly to illumination conditions during measurements and the biophysical characteristics of the canopy. The greater variation across cycles of measurements at different heights is observed for the shortwave downward flux of solar radiation (92%) and the lowest for the half-hourly wind speed standard deviation and proportion of areas with lower temperatures (3.95% for both).

Aiming to probe the dependence of reflectance on these control parameters, their bivariate analysis results are presented in Table A2. Bivariate analysis was conducted, including all data collected across all heights. Moderate and weak correlations between the proportion of areas with lower temperatures captured in thermal images AcAw and mean reflectance at NIR and SWIR indicate the importance of biophysical characteristics (*r* = −0.41 and −0.28 for NIR and SWIR, respectively).

Meteorological control parameters generally correlate very weakly with spectral reflectance mean values. The shortwave downward flux of solar radiation (*S_dw_*) positively correlates with reflectance in all tree wavelength bands (*r* = 0.44, 0.35, and 0.38 for the VIS, NIR, and SWIR bands of the spectrum); weaker correlations are observed for the wind speed variables. The effect of solar elevation changes on measured reflectance depends on the canopy characteristics or view geometry: i.e., in [61,62], it was suggested that for nadir-acquired reflectance factors, there was a strong solar angle dependence in all spectral bands: reflectance was found to increase for increasing solar elevation angles. Thus, a positive correlation between the shortwave downward flux of solar radiation and reflectance is plausible. For the measurements discussed here, a positive correlation between the shortwave downward flux of solar radiation (*S_dw_*) and the mean and standard deviation of wind speed has also been observed (*r* = 0.69 and 0.23 for *a* = 0.01 and 0.05, respectively); therefore, a clear causal relationship between the individual control parameters and reflectance is difficult to establish.

**Table A1:** Mean values of the parameters measured during Stage (i) of the top of the canopy spectral reflectance measurements.

	*h* = 1.75 m	*h* = 1.85 m	*h* = 1.95 m	*h* = 2.10 m	*h* = 2.15 m	*h* = 2.25 m	*h* = 2.50 m	*h* = 2.65 m
$\bar{\;R_{VIS}}\;$(%)	2.74	2.90	2.30	3.18	2.00	3.42	3.52	2.58
$\bar{\;R_{NIR}}\;$(%)	44.01	47.34	50.80	52.24	45.70	55.12	50.74	49.27
$\bar{\;R_{SWIR}}\;$(%)	11.54	12.48	11.15	13.92	10.26	14.32	12.73	11.35
$\bar{\;S_{dw}}$ (W m^−2^)	958.76	954.37	952.04	930.39	837.67	929.70	994.69	949.66
σSdw Sdw¯	2.05	0.46	2.59	0.25	0.32	0.19	3.33	3.70
$\bar{U}\;$ (m s^−1^)	2.48	2.55	2.90	2.20	1.95	2.29	2.52	2.97
$\sigma_{U}$ (m s^−1^)	1.27	1.23	1.31	1.16	1.24	1.18	1.27	1.24
AcAw	0.56	0.56	0.53	0.51	0.56	0.53	0.54	0.51
$\bar{T_{w}}\;$(°K*)*	22.81	23.31	27.16	21.76	23.33	21.34	20.18	25.27
$\min T_{w}$(°K)	15.91	16.08	23.14	14.56	19.49	14.39	13.83	20.45
$\max T_{w}\;$(°K)	47.49	48.37	41.83	45.28	35.54	44.98	37.98	38.44
$\bar{T_{c}}$ (°K)	14.93	15.43	22.89	14.75	19.28	14.41	14.01	20.76
$\min T_{c}\;$(°K)	10.91	11.37	20.27	11.04	17.15	10.55	9.50	16.78
$\max T_{c}$ (°K)	18.89	20.08	25.46	21.36	21.54	19.05	19.20	24.33

$\bar{\;R_{VIS}}$, $\bar{\;R_{NIR}}$, $\bar{\;R_{SWIR}}$ are the mean values of the top of the canopy reflectance for the VIS, NIR, and SWIR wavelength bands of the spectrum, respectively; AcAw is the proportion of areas with lower temperatures captured in thermal images; $\bar{\;S_{dw}}$ and σSdw Sdw¯ are the mean value and the coefficient of variation for the shortwave incoming solar radiation; $\bar{U}$ and $\sigma_{U}$ are the mean and standard deviation of the half-hourly wind speed during each cycle of measurements; $\bar{T_{c}}$, $\min T_{c}$, $\max T_{c}$ ($\bar{T_{w}}$, $\min T_{w}$, $\max T_{w}$) are mean, minimum, and maximum statistical values of the temperatures in the cooler (warmer) areas of the thermal images, respectively.

**Table A2:** Correlation coefficients between top of the canopy reflectance in the VIS, NIR, and SWIR wavelength bands and controlling variables during Stage (i) of measurements.

	$\bar{\;R_{VIS}}\;$ (%)	$\bar{\;R_{NIR}}\;$ (%)	$\bar{\;R_{SWIR}}\;$ (%)
$\bar{\;S_{dw}}\;$(W m^−2^)	0.44 *	0.35 *	0.38 *
σSdw Sdw¯	−0.10	−0.08	−0.19
$\bar{U}\;$ (m s^−1^)	0.12	0.20 **	0.06
$\sigma_{U}$ (m s^−1^)	−0.28 *	0.05	−0.23 **
AcAw	−0.20	−0.41 *	−0.28 *
$\bar{T_{w}}\;$ (°K)	−0.34 *	0.19	−0.15
$\min T_{w}$ (°K)	−0.57 *	0.07	−0.37 *
$\max T_{w}$ (°K)	0.41 *	0.14	0.37 *
$\bar{T_{c}}\;$ (°K)	−0.55 *	0.10	−0.37 *
$\min T_{c}\;$ (°K)	−0.65 *	0.03	−0.45 *
$\max T_{c}$ (°K)	−0.28 *	0.25 **	−0.14

$\bar{\;R_{VIS}}$, $\bar{\;R_{NIR}}$, $\bar{\;R_{SWIR}}$ are the mean values of the top of the canopy reflectance for the VIS, NIR, and SWIR wavelength bands of the spectrum, respectively; AcAw is the proportion of areas with lower temperatures captured in thermal images; $\bar{\;S_{dw}}$ and σSdw Sdw¯ are the mean value and the coefficient of variation for the shortwave incoming solar radiation; $\bar{U}$ and $\sigma_{U}$ are the mean and standard deviation of the half-hourly wind speed during each cycle of measurements; $\bar{T_{c}}$, $\min T_{c}$, $\max T_{c}$ ($\bar{T_{w}}$, $\min T_{w}$, $\max T_{w}$) are mean, minimum, and maximum statistical values of the temperatures in the cooler (warmer) areas of the thermal images, respectively. * *a* = 0.01 ** *a* = 0.05.

The dependence of spectral reflectance on the proportion of areas with lower temperatures captured in thermal images is more clearly depicted in Figure S8, where data were sorted according to AcAw ascending order and then binned in clusters of 10 points. Arranged in this way, the scatter is less, and the correlation coefficients increase to −0.59, −0.68, and −0.65 for VIS, NIR, and SWIR, respectively.

From the results discussed in this Section, it is evident that there is some variation in the spectral reflectance measurements conducted at different heights. There is also variation within the parameters that control the reflectance, which is related to illumination conditions and biophysical attributes associated with radiometric measurements. The next part of this Section deals with attempting to directly compare the spectral measurements conducted at different heights above the canopy while keeping the controlling parameters unchanged.

(b) Measurements taken at two heights: spectra comparison.

During stage (ii) of the canopy reflectance measurements, spectra were collected with the sensors placed at two heights only (2.10 m and 2.40 m above the mean canopy level). The mean reflectance values in the VIS, NIR, and SWIR wavelength bands are 3.5% (3.0%), 51.1% (46.4%), and 13.2% (12.0%) measured at 2.10 m (2.40 m) above the mean canopy height, respectively (Figure S9).

Bivariate analysis between spectral reflectance and control parameters measured during Stage ii is presented in Table S8. As was observed for the measurements taken during Stage (i), the proportion of areas with lower temperatures captured in thermal images AcoolAwarm correlates with the mean reflectance at NIR and SWIR (*r* = −0.54 and −0.58, respectively). Statistically significant positive correlations are observed between reflectance and statistical values of temperatures (mainly, the minimum temperatures for both the cooler and warmer areas in thermal images). It is hypothesised that this is related to both biophysical attributes and illumination conditions during the experiment, i.e., more sunlit, reflective surfaces lead to higher reflectance as well as surface temperatures, whilst more shadowed areas are related to lower reflectance and surface temperatures. Meteorological control parameters are only very weakly correlated with spectral reflectance mean values.

In order to compare the spectral measurements conducted at the two heights (*h* = 2.10 m and 2.40 m above the canopy), it is important to minimise the effect of the controlling variables, i.e., keep the controlling variables unchanged. The following steps have been taken:

- It has been observed that for this dataset, the controlling parameters which mostly affected the top of the canopy spectral reflectance are the proportion of areas with lower temperatures captured in thermal images (AcAw), and the minimum temperatures for both the cooler and warmer areas in thermal images ($\min T_{cool}$ and $\min T_{warm}$). Based on these results, a new variable has been created (*Reg R*) from the multiple linear regression of these control variables:
  (A1) RegR=a×AcAw+b×minTw+c×minTc+d

Using the data measured at both heights, the following results have been obtained: *a* = −22.4988, *b* = 4.9771, *c* = −1.1917, *d* = −13.1278

A scatter plot between the new regressed variable (*RegR*) and the mean reflectance measured at the NIR wavelength band is presented in Figure S10. The new *RegR* explains 81% of the observed variation in the reflectance data (*r* = 0.90).

- The new variable *RegR* ranged between 40.46 and 56.96, and each *RegR* value corresponded to two reflectance spectra measured at the two heights (2.10 m and 2.40 m, respectively). Data were segregated into seven clusters within this range: 40.46–45.41, 45.41–47.06, 47.06–4871, 48.71–50.36, 50.36–52.01, 52.01–53.66, 53.66–55.31, 55.31–56.96. All clusters had the same width (1.65), except the first (4.95) and the last one (3.3), so as to contain a sufficient number of datapoints for further analysis (Table A3). By segregating data in the way described here, it was made possible that within each cluster, variations of the control parameters would have as little effect as possible on the reflectance spectra.
- Within each cluster, the reflectance spectra at the two heights (2.10 m and 2.40 m above canopy level) were compared using the spectral separability method described in Section 2.5.1 By following the method described above, it was made possible that spectra measured at the two heights corresponded to similar conditions.

The results of this procedure are presented in Table A3. The spectral separability is generally very low in clusters 1–5 and 7 (average values are 1.2%, 0%, and 0.4% for the VIS, NIR, and SWIR, respectively). For cluster 7, spectral separability was very high (100%, 91.9%, and 76.9% for VIS, NIR, and SWIR, respectively). Even though bivariate analysis shows that for this dataset, the effect of meteorological control parameters does not significantly influence reflectance (Table S8), results found in the previous paragraph indicate that under certain conditions, their effect might be important. Therefore, in order to ensure that meteorological parameters remain unchanged within each cluster, the reflectance values corresponding to either shortwave downward flux of solar radiation outside the interquartile range or mean wind speed greater than 4 m s^−1^ have been filtered out. Results show that the overall spectral separability is very small (<1%); therefore, the effect of changing the height of sensors was negligible.

**Table A3:** Spectral separability at canopy level between measurements obtained at two heights (2.10 m and 2.40 m above mean canopy level) for three wavebands: visible (VIS: 300–700 nm), near-infrared (NIR: 750–1400 nm) and shortwave infrared (SWIR: 1400–2400 nm). The spectral separability reports the percentage of total bands that were significantly different (*a* = 0.01), as shown from two-sided Wilcoxon rank sum tests. In parenthesis, results after the effect of meteorological parameters (shortwave downward flux of solar radiation and wind speed) had been addressed.

Range of the Regressed Variable *		Number of Spectra		Spectral Separability (%)		
From	To	*h* = 2.10	*h* = 2.40	VIS	NIR	SWIR
40.46	45.41	11	15	5	0	1.4
		(5)	(4)	(0)	(0)	(0)
45.41	47.06	11	9	1.7	0	0
		(5)	(4)	(0)	(0)	(0)
47.06	48.71	14	8	0	0	0
		(6)	(3)	(0)	(0)	(0)
48.71	50.36	16	14	0.4	0	1.4
		(5)	(8)	(1.2)	(0.3)	(0)
50.36	52.01	15	15	100	91.9	76.9
		(6)	(4)	(7.5)	(0)	(6.8)
52.01	53.66	15	9	0	0	0
		(8)	(2)	(0)	(0)	(0)
53.66	56.96	17	8	0	0	0
		(8)	(2)	(0)	(0)	(0)

* Regressed variable (*Reg R*), obtained from the multiple linear regression of the control variables: AcAw, $\min T_{c}$, and $\min T_{w}$.
