# Supplementary material for: Exploring the Dependence of Spectral Properties on Canopy Temperature with Ground-Based Sensors: Implications for Synergies Between Remote-Sensing VSWIR and TIR Data
Source: Sensors (Basel). 2025 Feb 5;25(3):962. doi: 10.3390/s25030962 (PMC11820332; doi:10.3390/s25030962)
Supplement: Supplementary file 1 [file sensors-25-00962-s001.zip › sensors-3313271-supplementary.pdf]

Table S1: Leaf-level reflectance median values for three wavebands: visible (VIS: 300 nm – 700 nm), near infrared (NIR: 750 nm – 1400 nm) and shortwave infrared (SWIR: 1400 nm - 2400 nm).

Tree	Band	$\tilde{R}$	$IQR(R)$
1	VIS	8.5	8.7
	NIR	44.1	2.7
	SWIR	17.1	11.7
2	VIS	7.9	5.7
	NIR	47.4	2.1
	SWIR	17.3	13.7
3	VIS	5.7	4.2
	NIR	44.5	1.2
	SWIR	15.9	13.1
4	VIS	6.1	4.7
	NIR	43.8	1.7
	SWIR	16.8	12.8
5	VIS	10.1	7.4
	NIR	43.8	1.8
	SWIR	17.1	12.2
6	VIS	6.2	5.0
	NIR	47.0	1.7
	SWIR	19.1	13.0
7	VIS	7.9	7.6
	NIR	45.9	1.6
	SWIR	19.3	13.3

Table S2: Spectral separability at leaf-level between all trees for three wavebands: visible (VIS: 300 nm – 700 nm), near infrared (NIR: 750 nm – 1400 nm) and shortwave infrared (SWIR: 1400 nm – 2400 nm). The spectral separability reports the percentage of total bands that were significantly different (*a*= *0.01*) as shown from two-sided Wilcoxon rank sum tests.

Waveband	Trees	1	2	3	4	5	6
VIS	2	26					
	3	71	59				
	4	51	31	15			
	5	37	49	77	57		
	6	52	27	31	12	49	
	7	0	18	77	50	21	50
NIR	2	53					
	3	0	0				
	4	0	0	0			
	5	0	0	0	0		
	6	33	0	0	0	0	
	7	0	0	0	0	0	0
SWIR	2	0					
	3	35	0				
	4	0	0	0			
	5	0	40	31	0		
	6	89	0	100	59	61	
	7	0	0	57	0	0	0

Table S3: As in Table 1 but for the canopy reflectance (all measurements).

	D0			***σ***			***A***			***S***		
	Median	95th percentile	5th percentile	Median	95th percentile	5th percentile	Median	95th percentile	5th percentile	Median	95th percentile	5th percentile
Chlorophyll-1	0.52	0.60	0.45	103.60	105.20	96.00	48.38	56.43	40.25	2.24	2.42	2.14
Chlorophyll-2	0.91	0.94	0.88	140.40	148.70	132.10	116.62	126.83	109.16	2.59	2.72	2.35
Water -1	0.11	0.19	0.09	51.05	77.17	38.70	6.91	8.39	5.57	1.23	2.69	0.46
water -2	0.16	0.18	0.13	96.40	102.80	96.40	15.82	18.28	12.84	0.88	1.08	0.75
water -3	0.49	0.53	0.45	135.30	135.30	129.10	61.70	67.95	56.17	0.82	0.86	0.56
dry matter - 1	0.02	0.05	0.01	24.30	42.60	12.20	0.60	1.57	0.22	1.82	7.21	0.73
Water -4	0.15	0.21	0.10	79.30	103.80	52.97	12.52	18.44	6.91	1.23	3.17	0.69
water -5	0.75	0.81	0.69	167.30	179.70	154.90	119.79	132.55	106.89	0.42	0.61	0.28
Nitrogen	0.02	0.05	0.01	43.30	80.30	18.57	0.87	1.98	0.32	1.12	4.29	0.09
dry matter - 2	0.39	0.50	0.23	83.90	109.80	52.05	30.81	44.66	15.33	1.34	8.93	0.50

Table S4: As in Table 1, but for spectra corresponding to the third percentile of the A_c/A_w parameter.

	D0			***σ***			***A***			***S***		
	Median	95th percentile	5th percentile	Median	95th percentile	5th percentile	Median	95th percentile	5th percentile	Median	95th percentile	5th percentile
Chlorophyll-1	0.51	0.63	0.45	102.10	103.85	94.40	47.00	59.25	40.50	2.28	2.42	2.14
Chlorophyll-2	0.92	0.95	0.88	144.70	148.70	134.53	121.40	128.71	110.42	2.55	2.74	2.32
Water -1	0.13	0.19	0.09	45.00	76.70	38.68	7.05	8.39	5.98	1.99	2.70	0.51
water -2	0.16	0.18	0.14	96.40	102.80	95.75	15.99	18.75	12.85	0.90	1.13	0.81
water -3	0.49	0.53	0.45	135.20	135.90	128.49	61.31	67.81	55.68	0.80	0.87	0.55
dry matter - 1	0.02	0.06	0.01	21.30	42.60	6.10	0.63	1.37	0.18	1.97	8.55	0.70
Water -4	0.16	0.24	0.10	79.30	99.01	59.09	13.13	18.97	7.24	1.34	3.36	0.61
water -5	0.75	0.82	0.71	167.30	180.23	154.28	120.16	136.92	112.48	0.45	0.59	0.28
Nitrogen	0.03	0.06	0.01	49.50	81.01	17.97	1.09	2.78	0.49	1.15	3.59	0.08
dry matter - 2	0.41	0.51	0.20	86.90	108.61	41.24	32.90	45.74	13.30	1.43	11.97	0.58

Table S5: As in Table 1, but for spectra corresponding to the first percentile of the A_c/A_w parameter.

	D0			***σ***			***A***			***S***		
	Median	95th percentile	5th percentile	Median	95th percentile	5th percentile	Median	95th percentile	5th percentile	Median	95th percentile	5th percentile
Chlorophyll-1	0.54	0.59	0.45	108.00	111.10	106.50	53.02	58.00	42.45	2.48	2.79	2.33
Chlorophyll-2	0.91	0.93	0.88	140.20	145.80	132.08	116.15	125.25	109.26	2.78	2.87	2.71
Water -1	0.10	0.13	0.09	69.40	98.40	41.40	7.74	9.75	5.77	0.72	2.37	0.32
water -2	0.16	0.18	0.13	96.30	102.70	96.30	15.90	18.29	12.21	0.88	1.04	0.75
water -3	0.55	0.59	0.52	135.00	135.00	128.90	69.63	74.85	63.54	0.73	1.26	0.64
dry matter - 1	0.00	0.01	0.00	18.30	106.13	6.10	0.02	0.10	0.01	1.91	3.87	0.46
Water -4	0.11	0.16	0.06	73.30	85.68	39.85	7.51	12.35	3.29	1.42	3.02	0.73
water -5	0.74	0.80	0.69	148.80	161.20	142.60	109.21	117.83	96.79	0.47	0.72	0.30
Nitrogen	0.03	0.04	0.01	49.40	66.45	13.95	0.91	1.84	0.24	0.63	2.25	0.24
dry matter - 2	0.29	0.43	0.15	71.80	100.33	32.70	22.33	36.71	9.53	1.76	9.10	0.65

Table S6: Details of spectrometric measurements taken during the first stage of the experimental period (13/8/2019) at the cluster of trees.

Sensors' height (m) a.g.l.	Number of sets of measurements	Number of spectra and thermal images
5.10	3	9
5.18	3	9
5.29	4	12
5.47	3	9
5.50	8	48
5.59	3	9
5.66	3	9
5.85	3	9
6.03	8	24

Table S7: Details of spectrometric measurements taken during the second stage of the experimental period (17-20/9/2019) at the cluster of trees.

Sensors' height (m) a.g.l.	Date measurements were taken	Number of sets of measurements	Number of spectra and thermal images
5.47	17/9	10	66
	18/9	9	75
	19/9	5	43
	20/9	5	43
5.85	17/9	8	32
	18/9	10	50
	19/9	5	34
	20/9	5	32

Table S8. Correlation coefficients between top of the canopy reflectance in the VIS, NIR and SWIR wavelength bands, and controlling variables during Stage (ii) of measurements.

	$\overline{R_{VIS}}$ (%)	$\overline{R_{NIR}}$ (%)	$\overline{R_{SWIR}}$ (%)
$\overline{S_{dw}}$ $(W\,m^{-2})$	0.22	0.08	0.07
$\sigma_{S_{dw}} / \overline{S_{dw}}$	0.24	0.03	0.09
$\overline{U}$ $(m\,s^{-1})$	0.19	-0.15	-0.19
σ_U $(m\,s^{-1})$	0.13	-0.04	-0.13
A_c / A_w	-0.12	-0.54*	-0.58*
$\overline{T_w}$ (^oK)	0.07	0.47	0.18
$\min T_w$ (^oK)	0.53	0.77*	0.54*
$\max T_w$ (^oK)	0.11	0.23	0.20
$\overline{T_c}$ (^oK)	0.37	0.63*	0.36
$\min T_c$ (^oK)	0.57*	0.66*	0.64*
$\max T_c$ (^oK)	0.06	0.21	-0.09

$\overline{R_{VIS}}$, $\overline{R_{NIR}}$, $\overline{R_{SWIR}}$ are the mean values of the top of the canopy reflectance for the VIS, NIR and SWIR wavelength bands of the spectrum respectively; A_c / A_w is the proportion of areas with lower temperatures captured in thermal images; $\sigma_{S_{dw}} / \overline{S_{dw}}$ is the coefficient of variation for the shortwave incoming solar radiation; $\overline{U}$ and σ_U are the mean and standard deviation of the half hourly wind speed during each cycle of measurements; $\overline{T_c}$, $\min T_c$, $\max T_c$ ($\overline{T_w}$, $\min T_w$, $\max T_w$) are mean, minimum and maximum statistical values of the temperatures in the cooler (warmer) areas of the thermal images respectively.

* *a*=0.01 ** *a*=0.05

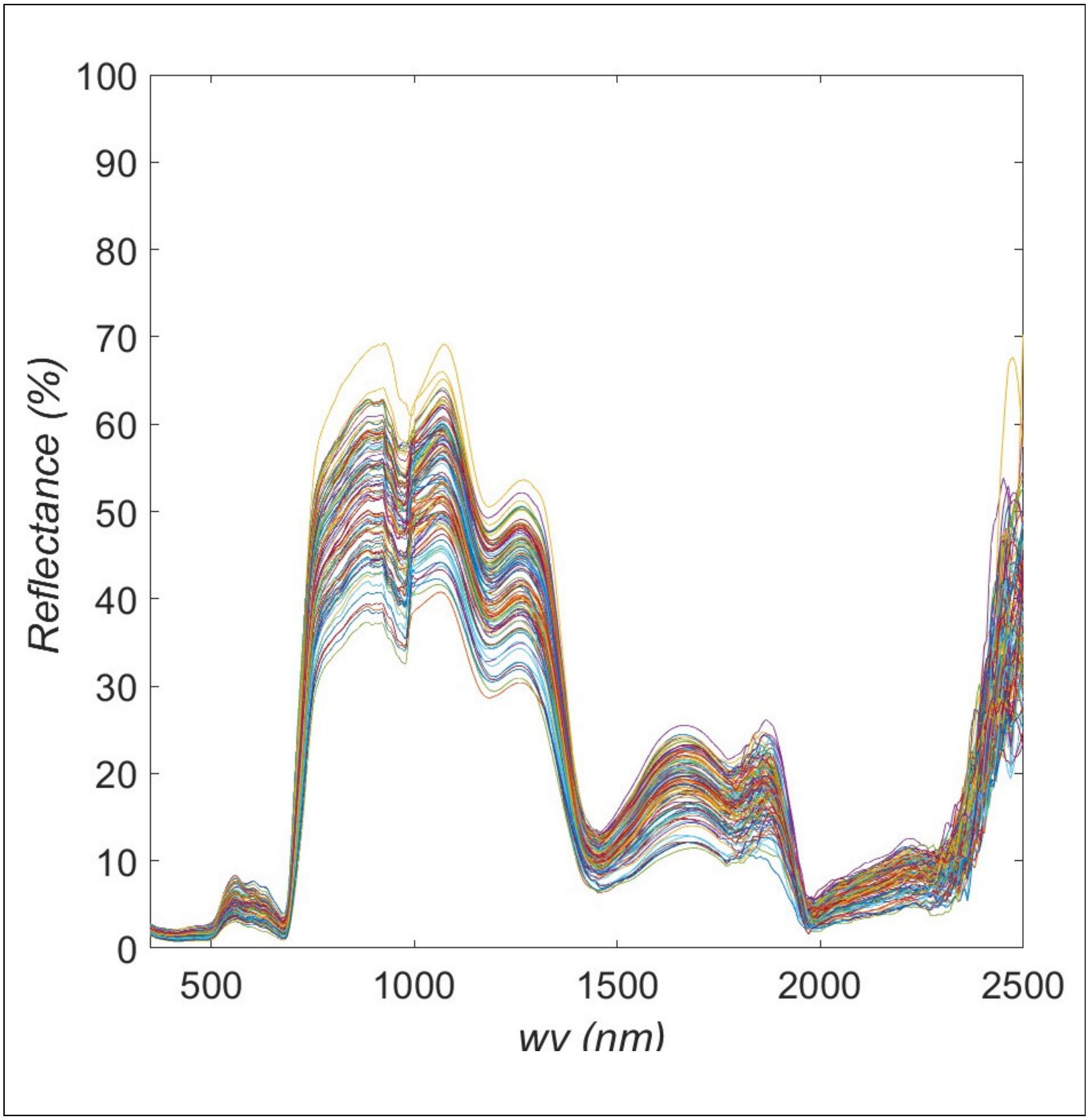

Figure S1: Raw spectral reflectance measurements at canopy scale used in this study.

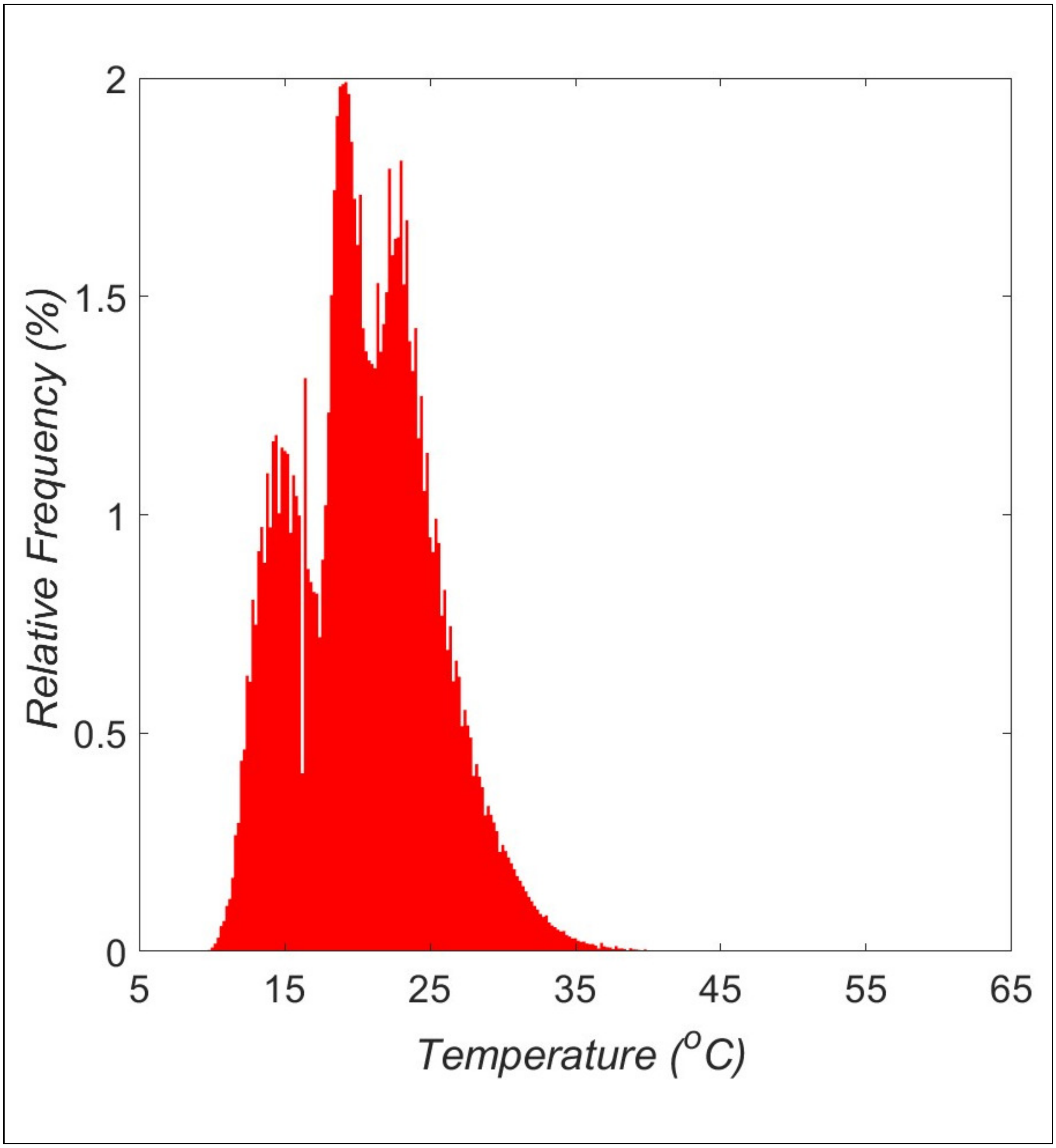

Figure S2: Raw surface temperature measurements at canopy scale used in this study.

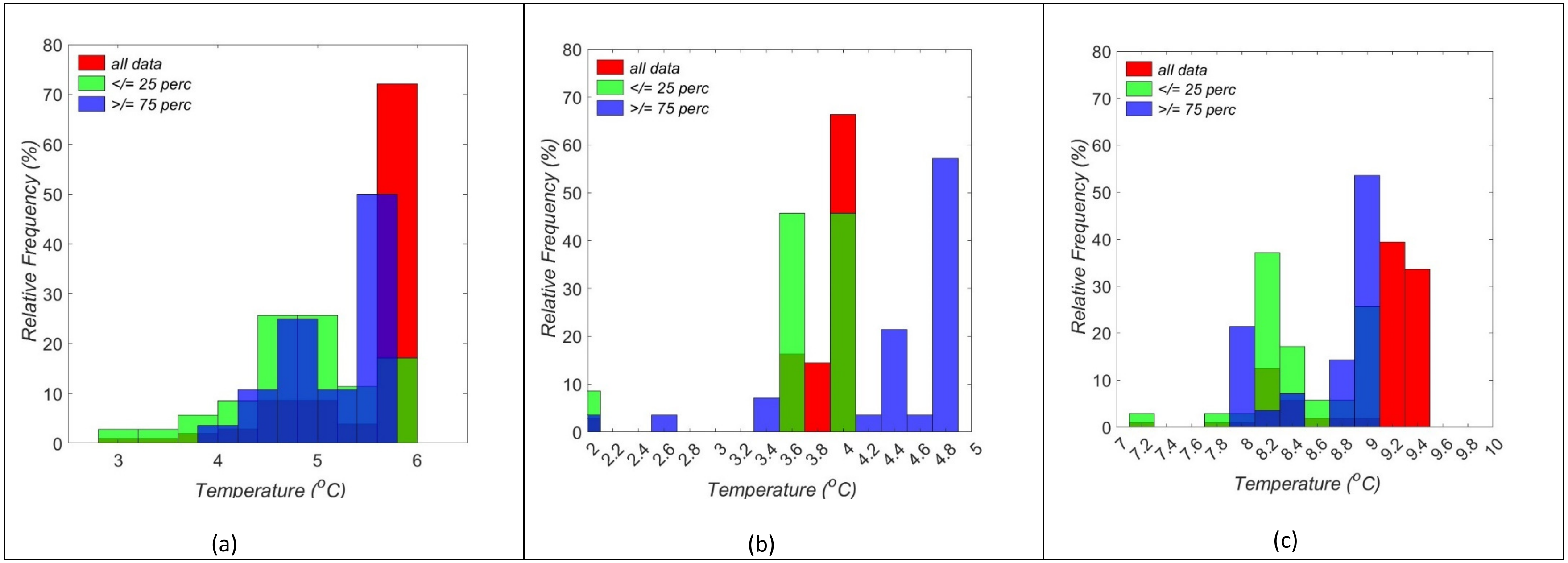

Figure S3: Histograms of relative frequency of the median (a), the lower (1st) (b) and higher (99th) (c) percentiles of the surface temperature difference between the cool and warm parts of the canopy for all values and values in the first and third quartile of the A_c/A_w parameter.

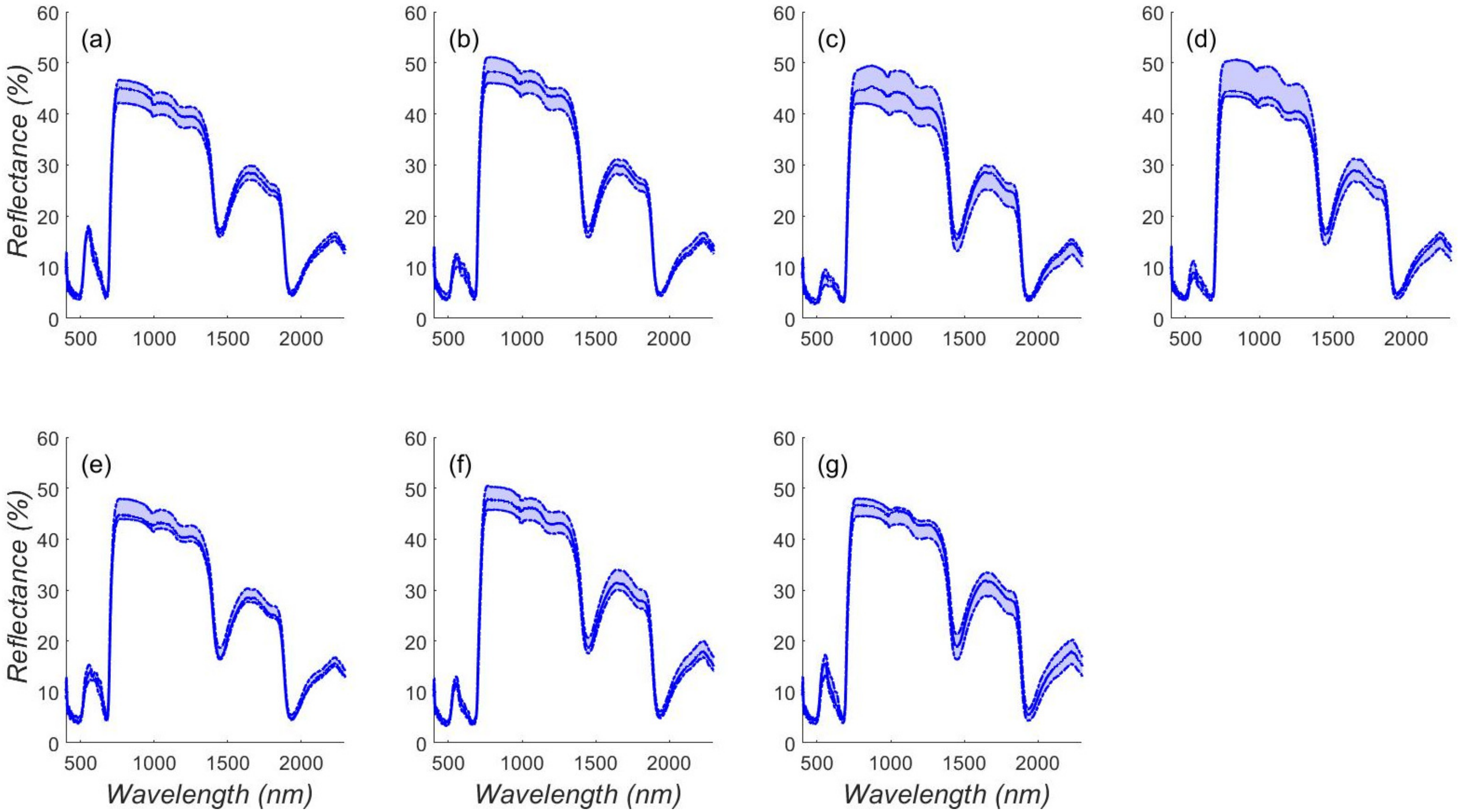

Figure S4: Composite plots of leaf-level reflectance spectra for Tree 1-7 (a – g respectively). Median reflectance is plotted as a solid thick line; interquartile range (25th – 75th percentiles) as dotted lines surrounding shaded areas

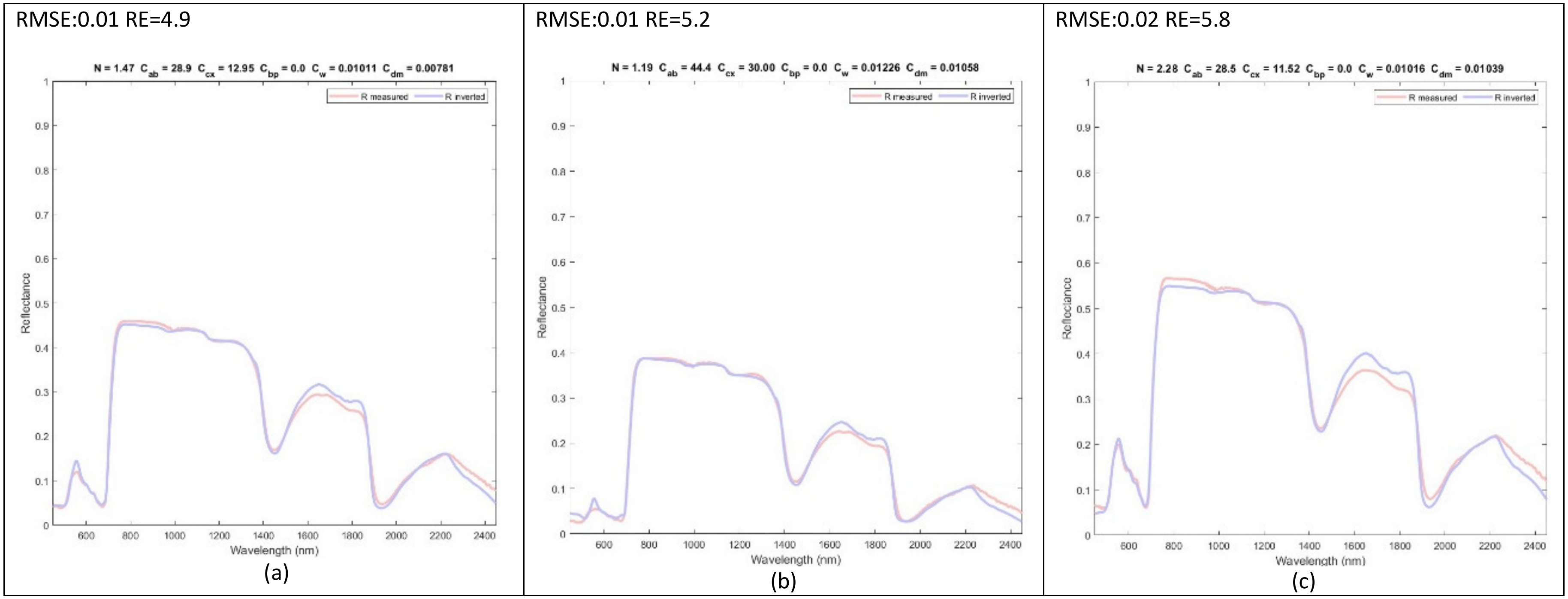

Figure S5: Comparison between leaf-level modeled (PROSPECT-5B) and measured reflectance spectra for the median (a) 1st and 99th percentile values of spectral reflectances measured at leaf-level. *N*: number of compact layers specifying the average number of air/cell walls interfaces within the mesophyll, C_{ab}: chlorophyll a+b content C_{ar}: carotenoids (carotenes + xanthophylls) content; C_{bp}: brown pigments content, C_w: equivalent water thickness; and C_{dm}: dry matter content. RMSE: Root Mean Square Error; RE: relative error (RMSE/mean).

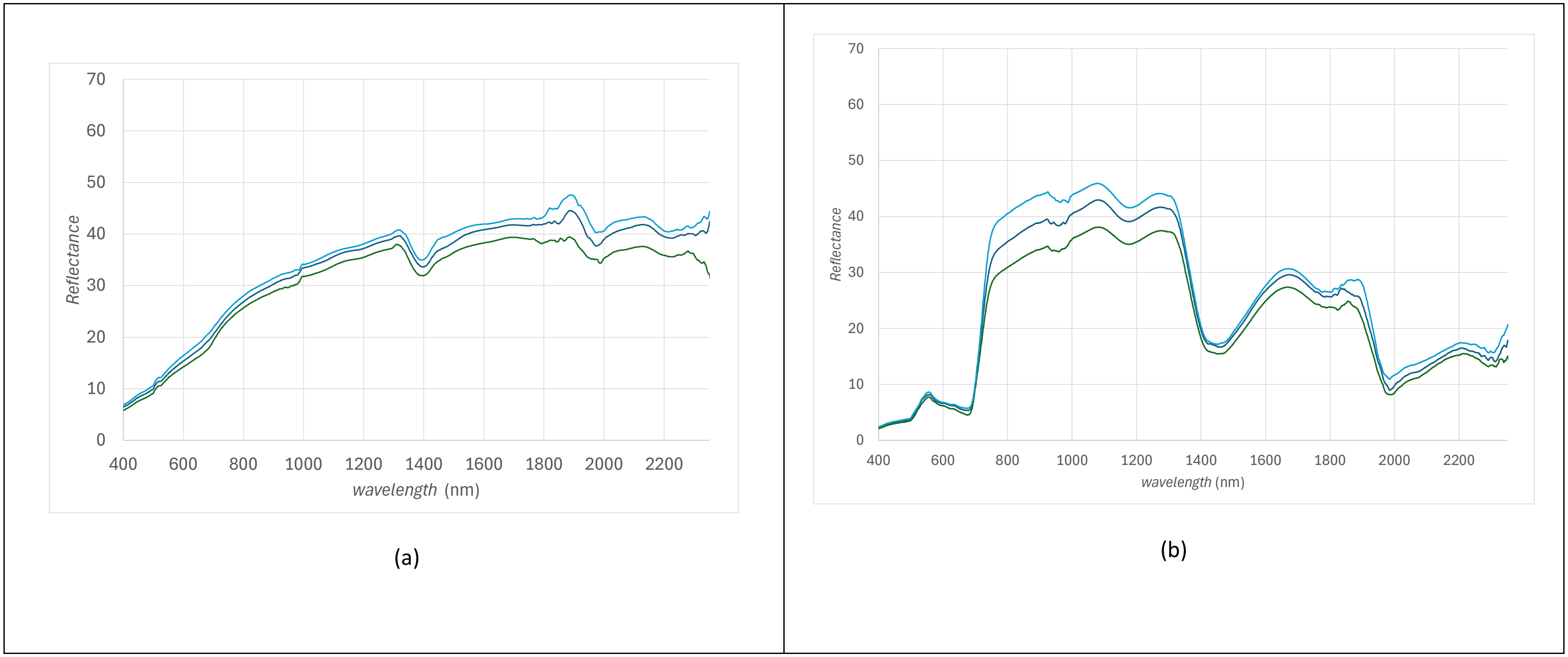

Figure S6: Measured soil (a) and grass (b) reflectance spectra.

RMSE=0.03 RE=13.0%

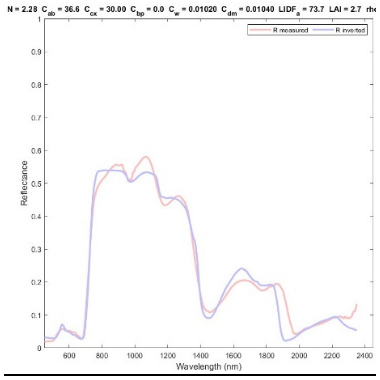

(a)

RMSE=0.02 RE=12.6%

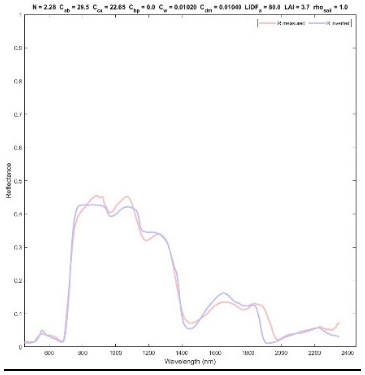

(b)

RMSE=0.04 RE=13.4%

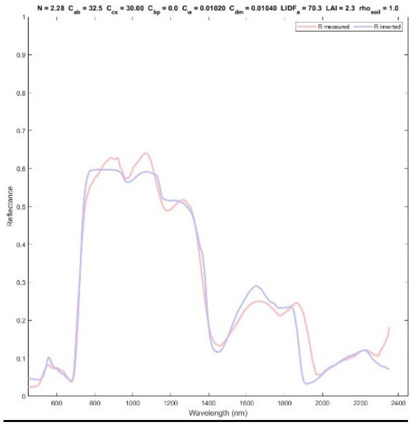

(c)

RMSE=0.03 RE=14.2%

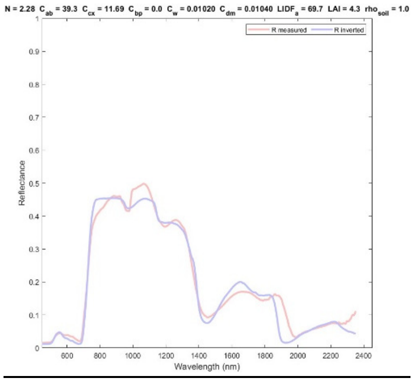

(d)

RMSE=0.02 RE=14.1%

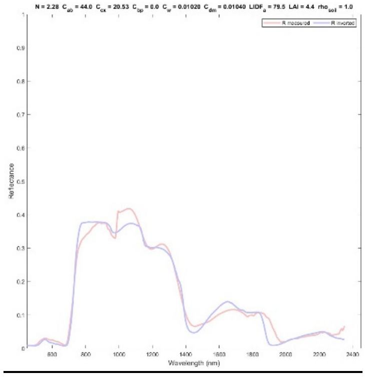

(e)

RMSE=0.04 RE=14.4%

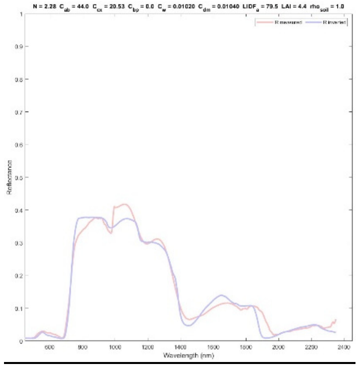

(f)

Figure S7: Comparison between leaf-level modeled (PROSPECT-5) and measured reflectance spectra for the median (a) 1st and 99th percentile values of spectral reflectances. *N*: number of compact layers specifying the average number of air/cell walls interfaces within the mesophyll, C_{ab}: chlorophyll a+b content C_{ar}: carotenoids (carotenes + xanthophylls) content; C_{bp}: brown pigments content, C_w: equivalent water thickness; and C_{dm}: dry matter content. RMSE: Root Mean Square Error; RE: relative error (RMSE/mean).

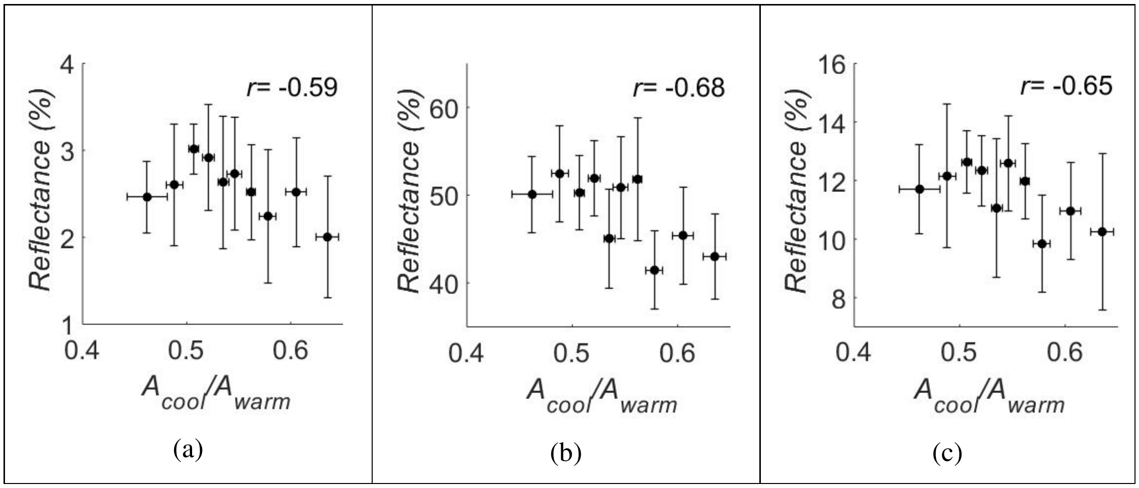

Figure S8: Scatter plots between sorted and binned values of the proportion of areas with lower temperatures captured in thermal images ($^{A_c}/_{A_w}$) and mean reflectance at the VIS (a), NIR (b) and SWIR (c) wavelength bands. The data were sorted according to $^{A_c}/_{A_w}$ ascending order and then binned in clusters of 10 points. The data shown are the mean values of $^{A_c}/_{A_w}$ and $\overline{R_{VIS}}$, $\overline{R_{NIR}}$ and $\overline{R_{SWIR}}$ within each one of these bins.

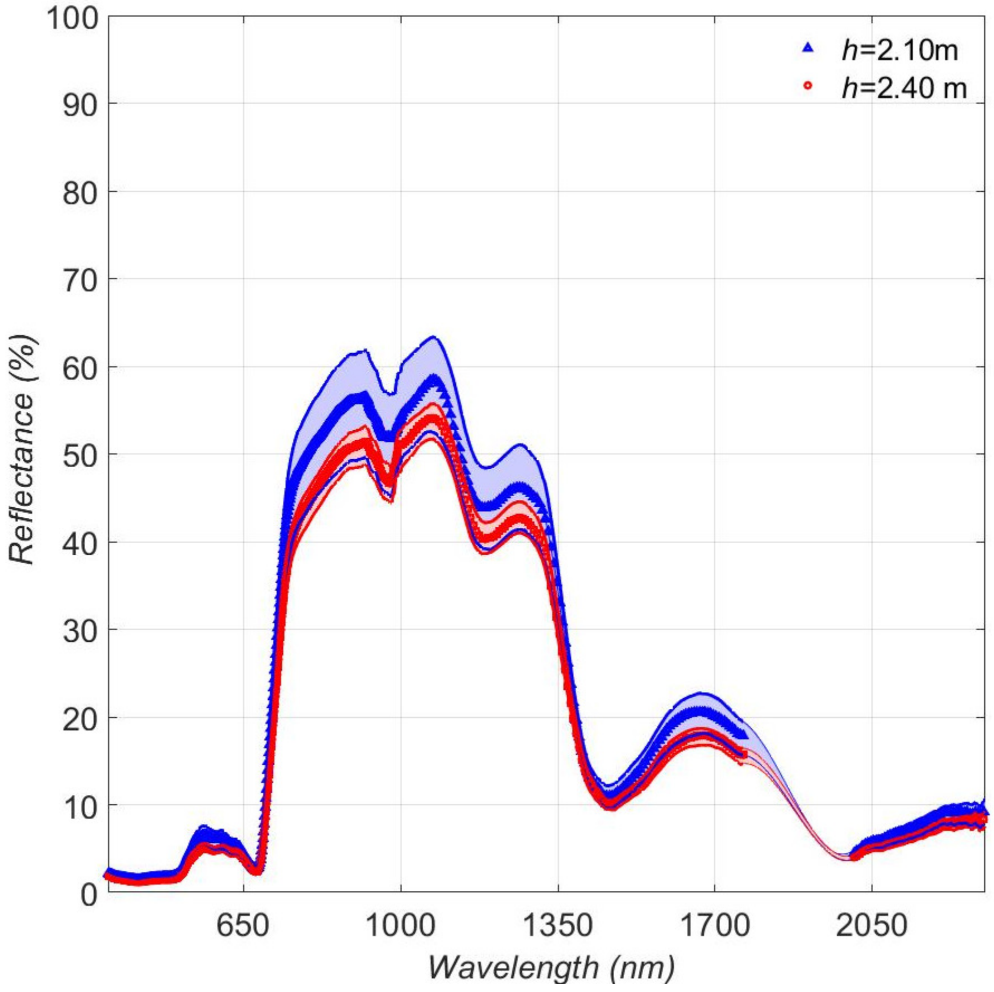

Figure S9: Composite plot of top of the canopy spectral reflectance measurements (Stage ii). Median reflectance is plotted as a solid thick line; interquartile range (25th – 75th percentiles) as shaded areas. *h*: height of the sensors above mean canopy level.

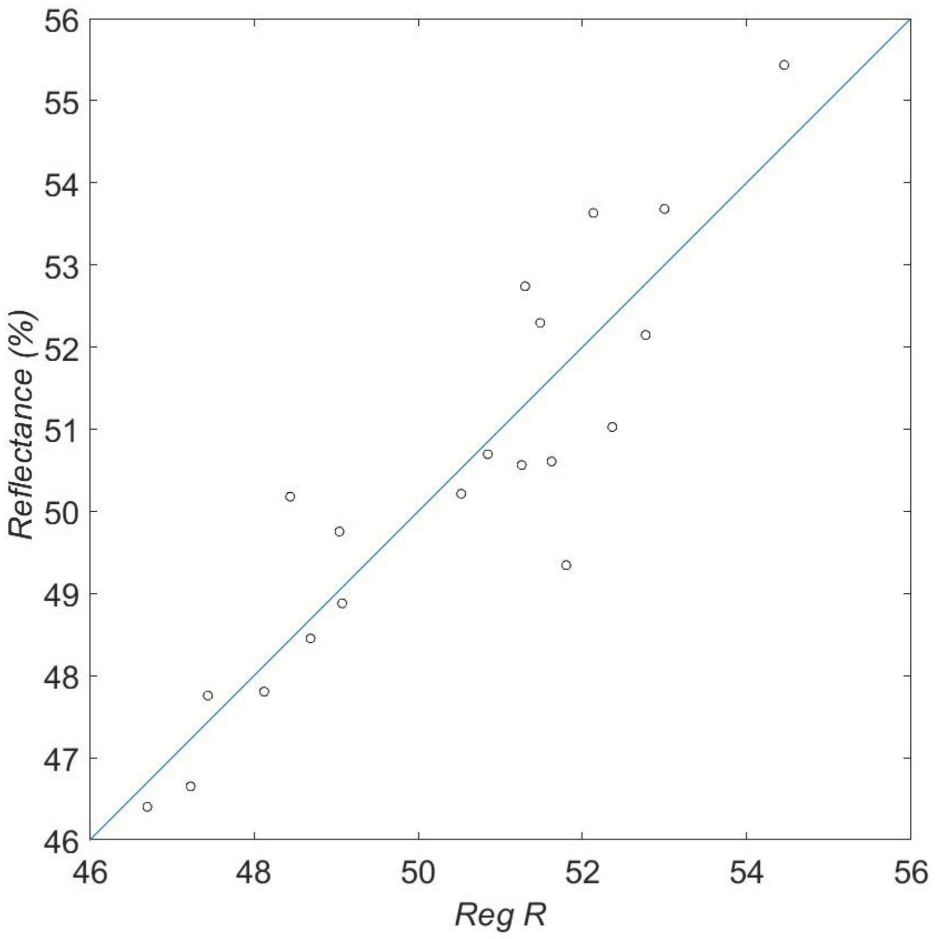

Figure S11: Scatter plot of binned mean reflectance at the NIR wavelength band and the regressed variable *RegR*. The presented data are the mean values within bins of ten datapoints of measurements. The blue line is the 1:1 line.
